# Antibacterial and photocatalytic potential of piperine-derived zinc oxide nanoparticles against multi-drug-resistant non-typhoidal *Salmonella* spp.

**DOI:** 10.1186/s12866-025-03829-4

**Published:** 2025-02-25

**Authors:** Varsha Unni, Padikkamannil Abishad, Bibin Mohan, Pokkittath Radhakrishnan Arya, Sanis Juliet, Lijo John, Valil Kunjukunju Vinod, Asha Karthikeyan, Nitin Vasantrao Kurkure, Sukhadeo Baliram Barbuddhe, Deepak Bhiwa Rawool, Jess Vergis

**Affiliations:** 1 Department of Veterinary Public Health, College of Veterinary and Animal Sciences, Pookode, KVASU, Kerala Veterinary and Animal Sciences University, Wayanad, 673 576 India; 2 Department of Veterinary Pharmacology and Toxicology, College of Veterinary and Animal Sciences, Pookode, Kerala Veterinary and Animal Sciences University, Wayanad, 673 576 India; 3 Department of Veterinary Biochemistry, College of Veterinary and Animal Sciences, Pookode, Kerala Veterinary and Animal Sciences University, Wayanad, 673 576 India; 4 Department of Veterinary Pathology, Nagpur Veterinary College, Nagpur, 440 006 India; 5 ICAR- National Meat Research Institute, Hyderabad, 500 092 India

**Keywords:** Antimicrobial resistance, Dye degradation, Green synthesis, Nanoparticle, Photocatalysis, Piperine, Zinc oxide

## Abstract

**Background:**

Drug-resistant pathogens and industrial dye wastes have emerged as critical global public health concerns, posing significant risks to human and animal health, as well as to environmental sustainability. Green synthesized nano absorbents were found to be a viable strategy for treating drug-resistant pathogens and in wastewater. Hence, this study endeavored the synthesis of piperine-driven nano-zinc oxide (ZnONPs) and evaluated them for antibacterial, antibiofilm, and photocatalytic disinfection potential against multi-drug resistant (MDR) foodborne strains of non-typhoidal *Salmonella* (NTS). Besides, the dye degradation potential of ZnONPs when exposed to UV, sunlight, and LED lights and their antioxidant capacity were assessed.

**Results:**

Initially, in silico analysis of piperine revealed drug-likeliness with minimal toxicity and strong interaction between piperine and OmpC motifs of *Salmonella* spp. UV spectroscopy of ZnONPs revealed a prominent absorption peak at 340 nm, while PXRD analysis confirmed the hexagonal wurtzite structure of ZnONPs by exhibiting peaks at 30°, 35.6°, 41.3°, 43.6°, 44.3°, 48°, 53°, 58°, and 59.2°, which corresponded to the lattice planes (102), (110), (103), (200), (112), (004), (104), (210), and (211). Additionally, the TEM images demonstrated predominantly spherical ZnONPs with hexagonal wurtzite crystalline SAED pattern. The minimum inhibitory concentration and minimum bactericidal concentration values (µg/mL) of the ZnONPs were found to be 62.50 and 125, respectively. The ZnONPs were observed to be safe with minimal hemolysis (less than 2%) in chicken RBCs, and no cytopathic effects were observed in the MTT assay using HEK cell lines. The NPs were found to be variably stable (high-end temperatures, proteases, cationic salts, and diverse pH), and were tested safe towards commensal gut lactobacilli. Additionally, in vitro time-kill kinetic assay indicated that the MDR-NTS strains were eliminated after co-incubating with ZnONPs for 6 h. The photocatalytic studies exhibited complete bacterial elimination under visible light at 4 h. Interestingly, the ZnONPs significantly inhibited the biofilm formation in the crystal violet staining assay by MDR-NTS strains (*P* < 0.001) at 24 and 48 h. Besides, a dose-dependent reducing power assay and 2,2′- azinobis (3-ethylbenzothiazoline-6-sulfonic acid) (ABTS^•+^) assay were exhibited. Moreover, ZnONPs significantly degraded methylene blue, crystal violet, and rhodamine-B under different light sources (sunlight, UV light, and LED).

**Conclusions:**

This study revealed a sustainable one-pot method of synthesizing ZnONPs from piperine, which might be used as a viable antibacterial candidate with antioxidant, antibiofilm, and photocatalytic properties with eco-friendly implications and wastewater treatment.

**Supplementary Information:**

The online version contains supplementary material available at 10.1186/s12866-025-03829-4.

## Background

Environmental pollution has become a major socio-economic threat to the global economy. Rampant urbanization and technological development have put life on Earth under threat by degrading the quality of vital elements, including water. Lately, water pollution associated with drug-resistant microbes and industrial wastes (including dyes) has received wide attention [1]. Owing to the indiscriminate use of antibiotics in various spheres, antimicrobial resistance (AMR) and drug-resistant pathogens are gaining alarming significance. AMR poses a grave global health challenge, leading to approximately 1.27 million deaths in 2019 and contributing to 4.95 million deaths. The primary drivers of AMR are the misuse and overuse of antimicrobials across humans, animals, and plants, impacting nations irrespective of their income levels. If no effective measures are taken, AMR is anticipated to result in 39 million deaths by 2050, with projected economic repercussions amounting to USD 1 trillion in additional healthcare costs [2]. Of late, drug resistance among foodborne pathogens has become an important public health threat. Non-typhoidal salmonellosis (NTS) is a major foodborne hazard that causes severe gastroenteritis and systemic illness, and it continues to be a significant global burden. Annually, NTS is responsible for an estimated 93.80 million cases of acute gastroenteritis globally, resulting in approximately 1,55,000 fatalities. The preponderance of such incidents is linked to consuming foods contaminated with NTS, particularly poultry and poultry-linked products [3, 4] Moreover, irrigation water poses a potential risk as a reservoir for drug-resistant pathogens, notably *Salmonella*, thereby contributing to contamination and serving as a vehicle for transmission [5]. Although NTS continues to significantly contribute to foodborne illness in most nations, the issue has been worsened by the sharp decline in the availability of antimicrobials for therapeutic use in public health practice [6]. Additionally, the promiscuous use of available antibiotics led to the development of multi-drug resistance (MDR) in NTS strains. In addition, dyes such as methylene blue (MB), crystal violet (CV), and rhodamine-B (RhB) are frequently employed in industries for the production of various polymers, cosmetics, medicines, and food [7]. These dyes are highly stable, do not readily degrade in water, and resist biodegradation [8]. This scenario has made it challenging to control pollution (drug-resistant pathogens and dyes), which has prompted scientists and researchers around the globe to consider suitable alternative strategies to halt their rapid emergence [9].

Nanoparticles (NPs) have recently been identified as a versatile tool and a viable strategy for treating drug-resistant pathogens associated with food and wastewater. Owing to their distinctive attributes (increased surface area-to-volume ratio, size, and charge), metal and metal oxide NPs have drawn considerable research attention worldwide as promising antimicrobial candidates and adsorbents for wastewater treatment [10]. Zinc oxide (ZnO) NPs have recently piqued researchers’ interest due to their chemical versatility and broad range of biological applications. In addition, ZnO NPs were generally recognized as safe (GRAS) by the United States Food and Drug Administration (US FDA) [11]. Because of their non-hazardous and non-toxic nature, ZnO NPs are well suited for various applications in photocatalysis, gene therapy, environmental remediation, water purification, and antimicrobial defense. The hyperkinetic activity of the ZnO NPs in the presence of light sources could be explored for their potential photocatalytic capabilities, paving the way for their applications in environmental remediation [12]. Although there are several modes of synthesizing NPs, the green synthesis method is much simpler, safer, more reliable, and biocompatible than physical and chemical techniques. Additionally, greener routes in fabricating the NPs have shown enhanced structural/morphological and biological properties [13, 14]. Conventional methods also necessitate the removal of toxic residues that can pose serious threats to the environment and public health, in addition to being more time-consuming [15]; thus, plant-based facile synthesis of NPs has been widely used.

Long pepper (*Piper longum* L.), acknowledged as Indian long pepper, is a deciduous aromatic climber that has extensively been used in traditional medicine for treating a variety of ailments because of its unique hepatoprotective, antioxidant, antitumor, antimicrobial, anti-dysenteric, immunomodulatory, anti-inflammatory, and anti-platelet properties [16]. The plant contains various bioactive compounds, including vicenin, volatile oils, anthraglycosides, tannins, saponins, sterols, alkaloids, piperine, and many others [17]. One of the most prominent alkaloids among plants of the Piperaceae family, especially *P. nigrum* and *P. longum*, is piperine. Piperine has been found to have analgesic, antidepressant, anticancer, antibacterial, and antitumor activities [18] and has recently received much attention due to its low toxicity [17]. Piperine has also been demonstrated to enhance the accumulation of reactive oxygen species (ROS), influence cell surface hydrophobicity, inhibit bacterial efflux pumps, and disrupt quorum sensing. It also possesses the ability to prevent initial adhesion, impede the maintenance of biofilms, and modulate the expression of biofilm-forming genes in bacteria [19–21]. Such low-toxic molecules could be devised as capping and reducing agents for the green synthesis of nano-ZnO. Several reports have documented the synthesis and antibacterial properties of ZnO NPs against *Salmonella* spp. [22, 23] and nano-ZnO from *P. longum* and piperine [24–26]. However, this work appears to be the first of its kind to explore the use of piperine in synthesizing ZnO NPs to evaluate the antibacterial and anti-biofilm efficacy against MDR strains of *S. enterica* Typhimurium and *S*. Enteritidis*,* which are among the most critical NTS strains causing global foodborne illnesses. Besides, our study provides detailed insights into the antibacterial effects and photocatalytic potential of piperine-capped ZnO NPs, presenting them as multifunctional agents for combating MDR pathogens and environmental decontamination. In light of these facts, this study is intended to investigate the in vitro antibacterial, antibiofilm, and photocatalytic disinfection potentials of biofabricated ZnO NPs synthesized with piperine, specifically targeting MDR strains of *S.* Typhimurium and *S.* Enteritidis. This study also includes the characterization of the ZnO NPs, their stability, safety, and antioxidant properties. Additionally, the photocatalytic dye degradation performance of these nanoparticles was evaluated, providing a comprehensive understanding of their potential for both pathogen control and environmental applications.

## Methods

### Bacterial strains

The MDR bacterial strains used in this study were isolated from poultry farm settings and stored in the laboratory repository (*S*. Enteritidis [S1; S2; S3] and *S*. Typhimurium [ST1; ST2; ST3]). The antimicrobial susceptibility testing [27] and PCR [28] assays were used to re-validate the strains (Supplementary Table 1). *E. coli* ATCC 25922 was used as the quality control strain for antimicrobial susceptibility testing.

### Chemicals and reagents

Piperine (≥ 97% purity) and analytical grade Zinc acetate dihydrate used in this study were procured from Sigma-Aldrich Pvt. Ltd., USA, while resazurin and dehydrated culture media were procured from HiMedia Laboratories Pvt. Ltd., Mumbai, India.

### In silico absorption, distribution, metabolism, excretion, and toxicity (ADMET) of piperine

Initially, a literature review using Google Scholar was performed to evaluate the antibacterial potential of piperine. Piperine is a single, well-characterized compound with inherent antimicrobial and antioxidant activities, which can synergistically enhance the functionality of the synthesized nanoparticles. The piperine in *P. longum* was subjected to an ADMET analysis using the Swiss ADME software (http://www.swissadme.ch/index.php). Further, the LD_50_ (mg/kg) and toxicity class of piperine were predicted using Protox-II (https://tox-new.charite.de/protox_II/ [29], whereas StopTox (https://stoptox.mml.unc.edu/) predicted cardiotoxicity [30].

### In silico molecular docking of piperine

A blind docking employing Autodock v.4.20 was carried out (Supplementary File, S1) to assess the binding affinity of piperine to ompC motifs of *Salmonella* spp. [31]. By eliminating the ligand, the water molecule, the hetero atoms, and the co-crystallized solvents, the target proteins were generated. Polar-charged hydrogen was added along with Gasteiger charges and non-polar hydrogens were combined. Besides, a grid map with 60 × 60x60 points and a 0.375 Å spacing was produced. The docking probability was further examined using the Lamarckian genetic algorithm. The ten best poses generated by the configuration files for OmpC motifs with piperine were recorded using the software. The ligand was scored according to docked energy, and molecular docking was shown using a Pymol viewer.

### Biosynthesis and characterization of ZnO NPs

Piperine (10 mg/mL in ethanol) was employed to fabricate ZnO NPs from 0.10 M of zinc acetate dihydrate solution. In brief, 70 mL of 0.10 M zinc acetate solution was mixed with 30 mL of piperine under constant stirring (300 rpm) at 60 °C for 2 h. The synthesis of ZnO NPs was evidenced by the color change of the reaction mixture from colorless to white. The ZnO NPs formed were subsequently washed thrice with methanol (Loba Chemie, India) and Milli-Q water; air-dried overnight at 80 °C and stored at 4 °C until use [26].

Initially, a UV–Vis spectrophotometer (ThermoFisher Scientific, USA) was used to scan the ZnO NPs between 250 and 450 nm after dissolving them in ultrapure water (1 mg/mL), whereas powder X-ray diffraction (PXRD; Bruker D8 Advance, USA) was used to perform structural analyses of the ZnO NPs at CuK radiation (40 keV, 40 mA, 0.02° scanning step size, ƛ = 1.54060 Å). Meanwhile, transmission electron microscopy (TEM) analysis of the samples was used to examine the shape and size of ZnO NPs (JEM 2100, Jeol, Japan).

### Determination of minimum inhibitory concentration (MIC) and minimum bactericidal concentration (MBC) of ZnO NPs

By assessing the MIC and MBC values of ZnO NPs against the test strains of MDR- S. Typhimurium and *S*. Enteritidis using the micro-broth dilution technique, the in vitro antimicrobial activity was determined [27].

In brief, the individual test cultures of MDR- NTS strains (100 µL; 1 × 10^7^ CFU/mL) were co-incubated on a 96-well flat-bottom microtiter plate with varying concentrations of ZnO NPs ranging from 500 to 0.244 mg/mL in 100 µL of cation-adjusted Mueller Hinton broth (CA-MH; HiMedia Laboratories Pvt. Ltd., Mumbai, India). Appropriate controls, including media control, solvent control, and respective test bacterial control, were employed in this assay. Following an 18–24 h incubation at 37 °C, 0.015% of resazurin dye was added to each well to determine the color change from purple to pink, indicating the degree of inhibition of bacterial strains.

The MIC value was determined as the least concentration of ZnO NPs without exhibiting visible growth, whereas the MBC was calculated by using the inoculum (10 µL) drawn from those wells displaying no visible bacterial growth and plating on selective agar (Xylose Lysine Deoxycholate [XLD], HiMedia) supplemented with ampicillin [32]. The MBC of ZnO NPs was determined to be the lowest dose that was required to kill MDR-NTS strains on selective agar by 99.90% [33].

### In vitro stability and safety assays of ZnO NPs

The stability of ZnO NPs synthesized using piperine as effective therapeutic molecules (Supplementary File, S1-S4) was comparatively evaluated by exposing them to high temperatures (70 °C; 90 °C), proteases (trypsin, lysozyme, and proteinase- K), cationic salts at the physiological concentration (150 mM NaCl; 2 mM MgCl_2_) and varying pH (4.0, 6.0, and 8.0) [34].

Later, the safety of biofabricated ZnO NPs on the host cells was comparatively determined (Supplementary File, S5-S7) using a hemolytic assay in chicken erythrocytes and a cytotoxicity assay employing human epithelial embryonic kidney (HEK) cell lines as well as its effect on beneficial gut lactobacilli [34].

### In vitro antioxidant potential of biofabricated ZnO NPs

The reducing power assay [35] and 2,2′- azinobis (3-ethylbenzothiazoline-6-sulfonic acid) (ABTS• +) assay [36] were used to compare the antioxidant potential of ZnO NPs, keeping ascorbic acid as the control (Supplementary File, S8-S9).

### In vitro antibiofilm potential of ZnO NPs

Using the crystal violet staining assay in 96-well microtiter plates, the antibiofilm activity of biofabricated ZnO NPs was evaluated against the tested MDR-NTS strains both at 24 and 48 h [37]; Supplementary File, S10).

In brief, MDR-NTS strains (10^7^ CFU/mL; 50 µL) and ZnO NPs (50 µL) were co-incubated in sterile nutrient broth supplemented with 0.45% D-Glucose (HiMedia) with suitable controls. The positive control consisted of respective untreated MDR-NTS strains (50 μL) in nutrient broth (50 μL), while the negative control consisted of sterile nutrient broth (100 μL). Besides, *E. coli* ATCC 25922 was used in this assay as a known biofilm-forming strain, and *E. coli* DH5α served as a non-biofilm former. To determine the inhibition of biofilm biomass, the absorbance at 595 nm was measured (Bio-Rad iMark Microplate reader, USA). The reduction in biofilm biomass over 24 h and 48 h was estimated as

$${\varvec{B}}{\varvec{i}}{\varvec{o}}{\varvec{f}}{\varvec{i}}{\varvec{l}}{\varvec{m}}\boldsymbol{ }{\varvec{r}}{\varvec{e}}{\varvec{d}}{\varvec{u}}{\varvec{c}}{\varvec{t}}{\varvec{i}}{\varvec{o}}{\varvec{n}}\boldsymbol{ }(\boldsymbol{\%})=\boldsymbol{ }\frac{{{\varvec{A}}}_{{\varvec{C}}{\varvec{o}}{\varvec{n}}{\varvec{t}}{\varvec{r}}{\varvec{o}}{\varvec{l}}}-\boldsymbol{ }{{\varvec{A}}}_{{\varvec{T}}{\varvec{e}}{\varvec{s}}{\varvec{t}}}}{{{\varvec{A}}}_{{\varvec{C}}{\varvec{o}}{\varvec{n}}{\varvec{t}}{\varvec{r}}{\varvec{o}}{\varvec{l}}}}\times 100$$

wherein A_Control_ denotes the absorbance of untreated control taken at 24 h and 48 h, whereas A_Test_ denotes the absorbance of treated samples taken at 24 h and 48 h.

### In vitro photocatalytic disinfection of MDR-NTS strains treated with ZnO NPs

The log-phase cultures of individual MDR- NTS strains (10^7^ CFU/mL; 100 µL) in CA-MH broth were co-incubated with the ZnO NPs (100 µL) at MIC and MBC levels to assess the in vitro dose- and time-dependent extracellular growth kinetics of MDR-NTS strains treated with biofabricated ZnO NPs. The treatment control employed in this study comprised corresponding MDR-NTS isolates treated with 10 µg/mL of meropenem, whereas the respective MDR-NTS isolates in sterile CA-MH broth were used as the untreated control. To investigate the in vitro photocatalytic growth kinetics, the individual MDR-test strains were treated with different concentrations (MIC, 1/2X MIC, 1/5X MIC and 1/10X MIC) of ZnO NPs on exposure to LED light (63,400 lx, 460 nm, 50 W [Opple, India]).

The bacterial enumeration was carried out using the aliquots drawn at specific time points (0, 30 min, 1 h, 2 h, 3 h, 4 h, 6 h, and 24 h), plated [34] on XLD agar supplemented with ampicillin, incubated at 37 °C for 24 h and were represented as log_10_CFU/mL.

### Photocatalytic degradation of cationic dyes treated with ZnO NPs

The ZnO NPs were treated with three different cationic dyes i.e., MB, CV, and RhB (Loba Chemie, India) on exposure to different light sources to assess the degradation capacity.

Briefly, the dyes (5 ppm for MB and RhB, 50 ppm for CV) were mixed separately with the ZnO NPs (0.50 mg/mL) in aqueous solution. The dye-NP mixtures were magnetically stirred in the dark at 300 rpm for 15 min to reach adsorption equilibrium. Subsequently, the mixtures were exposed to sunlight (11.5408348°, 76.0209177°; 52,100 lx), LED light (63,400 lx, 460 nm, 50 W), and UV light (260 nm, 16 W [Violight, India]) separately. Samples collected at specific time intervals (0, 15, 30, 45, 60, 75, 90, and 105 min) were centrifuged at 5000 rpm for 15 min and the absorbance was measured at 670, 592, and 550 nm for MB, CV, and RhB, respectively using a UV–Vis spectrophotometer (ThermoFisher Scientific, USA).

The dye concentrations were estimated from absorbance using the Beer-Lambert law [38], and the degradation was assessed [39] by,

$${\varvec{D}}{\varvec{e}}{\varvec{g}}{\varvec{r}}{\varvec{a}}{\varvec{d}}{\varvec{a}}{\varvec{t}}{\varvec{i}}{\varvec{o}}{\varvec{n}}\boldsymbol{ }(\boldsymbol{\%})=\boldsymbol{ }\frac{{{\varvec{C}}}_{0}-{{\varvec{C}}}_{1}}{{{\varvec{C}}}_{0}}\times 100=\frac{{{\varvec{A}}}_{0}-{{\varvec{A}}}_{1}}{{{\varvec{A}}}_{0}}\times 100$$

wherein C_0_ represents the initial concentration of the dye, C_1_ is the concentration of dye at a specific time point, A_0_ is the initial peak absorbance of the dye, and A_1_ is the peak absorbance at a given time point.

The photocatalytic reaction kinetics were quantified using different kinetic models (Supplementary File, S11).

### Statistical analysis

Each experiment was performed three times in triplicate and the results were analyzed using GraphPad Prism 8.2.1 (GraphPad Software Inc., San Diego, CA, USA) at 5% confidence intervals. To compare the differences between the cytotoxicity of control and ZnO NPs-treated cell lines and the antibiofilm potential of biofabricated ZnO NPs, a one-way analysis of variance (ANOVA) with Bonferroni multiple comparison post-test was used. The in vitro growth kinetics of MDR-NTS strains were analyzed using a two-way (repeated measurements) ANOVA with a Bonferroni multiple comparison post-test. *P*-values ≤ 0.05 were regarded as statistically significant, and those ≤ 0.01 as highly significant.

## Results and discussion

Nanotechnology has been acknowledged as one of the most important scientific developments in the struggle against drug-resistant bacteria [40]. Metal and metal oxide NPs are becoming increasingly popular [10], with ZnO NPs being widely used in biomedicine due to their biodegradability and low toxicity. The distinctive characteristics of ZnO NPs, such as their diminutive dimensions and huge surface area, have recently piqued the interest of researchers, making them a viable candidate for resolving the current AMR crisis and photocatalysis, including the bacterial disinfection and degradation of cationic dyes [26]. Numerous approaches to the synthesis of NPs have generally been put forth; using plant extracts is frequently a simple, dependable, sustainable, and environmentally friendly process [41–43]. The phytochemicals associated will provide improved antimicrobial properties [17]. This study employed the synthesis of ZnO NPs using piperine, their in vitro safety, stability, antioxidant, antimicrobial, antibiofilm, as well as photocatalytic disinfection potential against the MDR strains of foodborne NTS and the photocatalytic degradation of industrial cationic dyes on treatment with the ZnO NPs.

### In silico ADMET analysis and molecular docking

The ADME analysis (water solubility, lipophilicity, physicochemical properties, medicinal chemistry, drug-likeness, and pharmacokinetics) of piperine revealed drug-likeness without any violations of Lipinski’s rule of five (Supplementary Table 2) by Swiss ADME software. A bioavailability score of 0.55 indicated that piperine had drug-like properties; moreover, piperine was found to absorb in the gastrointestinal tract and could cross the blood–brain barrier. Besides, it was noticed that the consensus log Po/w (a measure of lipophilicity) was 3.03, and the topological polar surface area was 38.77 Å^2^. Additionally, the piperine showed non-permeability to the glycoprotein substrate (P-gp). Moreover, piperine was located largely inside the pink area of the bioavailability radar plots (Supplementary Fig. 1), suggesting the drug likelihood with a better bioavailability profile. In addition, the boiled egg graph of piperine exhibited a red point within the yellow (yolk) region (Supplementary Fig. 2), indicating that the compound penetrates the brain and may operate as a non-substrate of P-gp. Understanding the blood–brain barrier penetration helps in assessing the overall safety and potential off-target effects of ZnO NPs, especially if they are considered for medical applications beyond antimicrobial activity, such as in drug delivery systems. Moreover, the in silico toxicity characteristics of piperine assessed by Protox-II and StopTox software predicted an LD_50_ (mg/kg) of 330; as a result, piperine was categorized under toxicity class-4. With confidence levels of 63 and 72%, piperine was also predicted to be non-toxic when inhaled and applied topically (Supplementary Table 3).

**Fig. 1:**
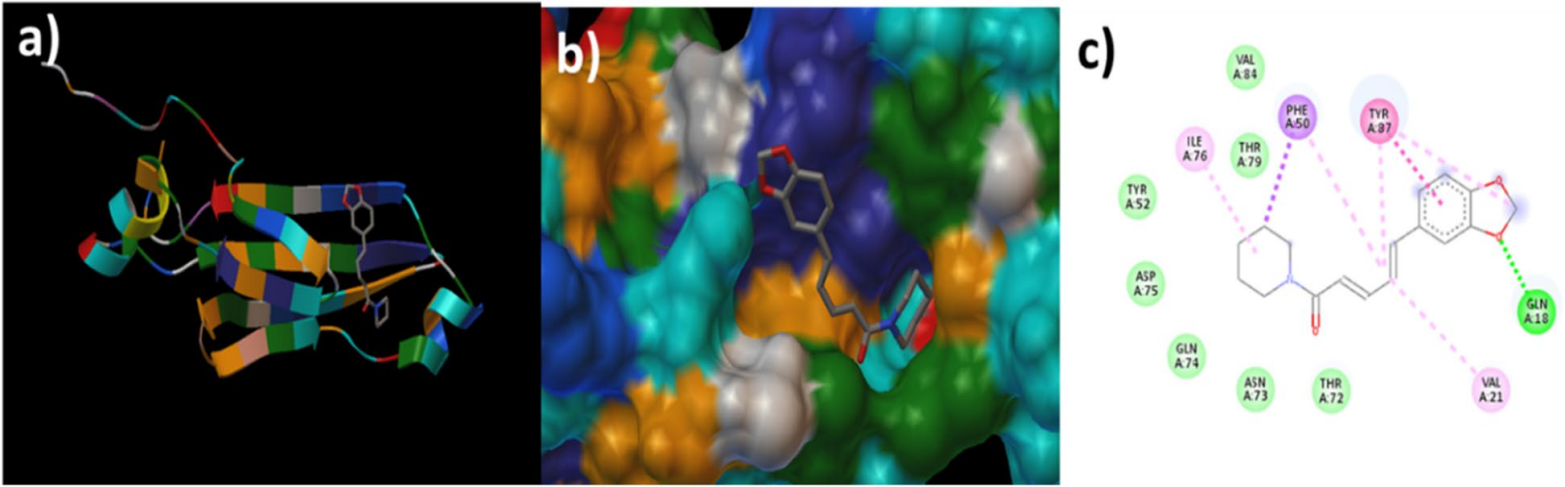
In silico molecular docking of piperine against ompC motif of *Salmonella* spp. Images denote the secondary structure of the piperine-ompC complex (**a**), the 3-D conformation of the piperine-ompC complex (**b**), and the piperine-ompC interaction (**c**)

The molecular docking was attempted using the constructed ligand structure of piperine on the outer membrane protein ompC of NTS strains (PDB ID: 3UU2) to evaluate their binding interactions. With a binding energy of −6.55 kcal/mol, piperine was bound to ompC (Supplementary Table 4). In addition, conventional hydrogen bonding was exhibited at active sites of GLN18, π-Σ hydrophobic interactions at the sites of PHE50, π-π stacked hydrophobic interactions at TYR87, alkyl hydrophobic interaction at VAL21 and ILE76, and π-alkyl bond at the sites of PHE50 and TYR87. Furthermore, van der Waal’s interaction was observed at THR72, ASN73, GLN74, ASP75, TYR52, THR79, and VAL84 (Fig. 1). It was concluded that piperine had a significant impact as a potential inhibitor and interacted strongly with the ompC motifs of *Salmonella* spp.

### Biosynthesis of ZnO NPs

*P. longum* is a well-known spice that has been used in medicinal preparations for centuries to treat a variety of ailments. Additionally, the bioactive elements present in the *P. longum* extracts like piperine serve as capping as well as reducing agents [26]. Moreover, piperine has been identified as the primary bioactive component in pippali and was reported to have numerous therapeutic benefits, including antimicrobial, antihypertensive, antioxidant, analgesic, anti-diarrhoeal, antidepressant, antiplatelet, and anticancerous properties. Additionally, piperine was documented to inhibit the growth of various MDR pathogens, including methicillin-resistant *Staphylococcus aureus*, *S*. Typhi, *E. coli*, and *Proteus* spp. [44]. In this study, commercial piperine was used to reduce 0.10 M zinc acetate dihydrate solution (1:4 ratio) to ZnO NPs at 60 °C for 2 h at room temperature with continual stirring. The synthesis of ZnO NPs was indicated by the development of a white precipitate at the bottom of the beaker. Piperine acted as a reducing agent during the synthesis of ZnO NPs, which might have resulted in a visible color change of the solution from colorless to white.

### Characterization of ZnO NPs

**Fig. 2:**
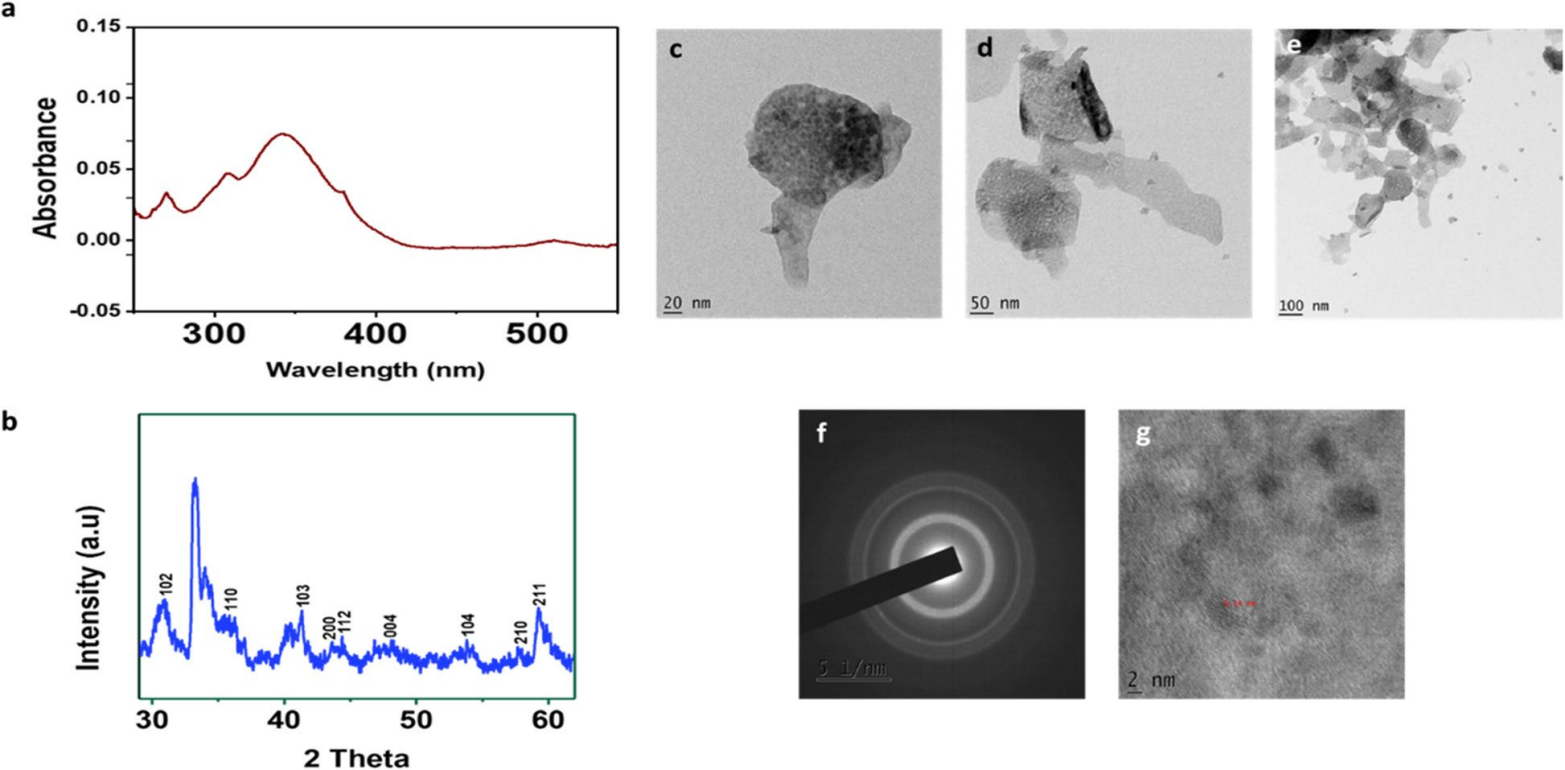
Physico-chemical characterization of green synthesized ZnO NPs. UV–Vis spectra (**a**), PXRD pattern (**b**), low- and high-magnification TEM images (**c**, **d**, **e**); SAED pattern (**f**) and Fourier filtered HR-TEM (g) images of green synthesized ZnO NPs

A progressive surface plasmon resonance (SPR) band in the wavelength range of 320–350 nm, with a maximum absorption peak at 340 nm, was visible in the UV visible spectra of the ZnO NPs created with piperine (Fig. 2a) [45]. The results indicated that piperine was essential in the reduction of Zn^2+^ (found in Zinc acetate) to Zn^0^ (ZnO NPs) ions [46].

The PXRD analysis performed to comprehend the crystalline nature and to confirm the formation of ZnO NPs [47] exhibited the peaks at 2θ values (30°, 35.6°, 41.3°, 43.6°, 44.3°, 48°, 53°, 58°, and 59.2°) (Fig. 2b), that corresponded to the lattice planes (102), (110), (103), (200), (112), (004), (104), (210), and (211). The entire peaks conformed to the Joint Committee on Powder Diffraction Standards (JCPDS) File No. PDF01-070–8070 [48]. The observed intense and sharp peaks in PXRD demonstrated that the biosynthesized ZnO NPs employing piperine exhibited a hexagonal wurtzite crystalline structure [49] as obtained in our previous study using *P. longum* catkin extract [26]. Additionally, the Debye–Scherrer formula estimated that the biofabricated ZnO NPs exhibited an average crystallite size of 32.8 nm.

The TEM images revealed that the piperine-mediated ZnO NPs were almost spherical with a minute alteration in thickness. Moreover, the agglomeration of ZnO NPs (Fig. 2c-e) could be due to the presence of piperine capped on the ZnO NPs [49]. Moreover, the SAED pattern of ZnO NPs demonstrated a hexagonal wurtzite crystallinity (Fig. 2f), which correlated well with the results of PXRD. Besides, a spacing of lattice fringes in the range of 0.24 nm was demonstrated by the TEM image of the piperine-mediated green synthesized ZnO NPs (Fig. 2g).

### In vitro determination of MIC and MBC

The biosynthesized ZnO NPs from piperine were reported to exhibit MIC and MBC values of 62.50 µg/mL and 125 µg/mL, respectively, against the tested MDR- NTS strains (Supplementary Table 5). The antibacterial properties of ZnO NPs might be attributed to the generation of reactive oxygen species and the presence of piperine. The interaction of ZnO NPs with bacterial membranes may lead to increased permeability or direct disruption of the lipid bilayer. This effect along with ROS compromises membrane integrity and permeability leading to the leakage of intracellular contents and ultimately bacterial death. The incorporation of piperine as a stabilizing and capping agent in the synthesis of ZnO NPs enhances their activity [50–52]. Piperine itself possesses antimicrobial properties, and its presence may amplify ROS generation and improve nanoparticle stability and dispersion, increasing their contact with bacterial cells. The in vitro killing kinetic assays could largely explain the antimicrobial properties.

### In vitro stability assays

#### Effect of high-end temperatures

Since animal feed processing involves several steps, such as mash preparation (45–55 °C), pelletization (around 80 °C), and steam sterilization at 100 °C, ZnO NPs should preferably be stable for their practical use as feed ingredients [53]; hence, the stability of green synthesized ZnO NPs was evaluated at high-end temperatures (70 and 90 °C) for specific time intervals (5, 15, and 30 min).

The MIC values of ZnO NPs synthesized from piperine remained unchanged at 70 °C for all strains, except ST2 and ST3 (Supplementary Table 6), and all strains exhibited constant MIC values when incubated at 90 °C. Nevertheless, the MBC values for all the strains were found to increase (2- to threefold times), both at 70 °C and 90 °C. In short, the green synthesized ZnO NPs were variably stable and capable of withstanding high-end feed processing temperatures [54]. Previous studies have highlighted the pivotal role of piperine in improving nanoparticle characteristics through surface modifications and stabilization. These enhancements likely contributed to preventing nanoparticle aggregation and mitigating thermal degradation when exposed to elevated temperatures [55, 56].

#### Effect of proteases

The surface of green synthesized NPs can change due to interaction with proteases, which can then cause aggregation and destabilization. Therefore, the NPs should withstand the action of a variety of proteases in order to be deemed stable. Furthermore, protease-mediated degradation may render the NPs inactive, affecting their therapeutic efficacy [57].

The MIC values for ZnO NPs synthesized from piperine were generally reduced by half (62.50 μg/mL) upon exposure to trypsin; however, strain-wise variation was noted (Supplementary Table 7). In addition, the MIC values of ZnO NPs treated with lysozyme remained constant for all the tested strains except ST2 and ST3, which were observed to have doubled. Interestingly, the MIC values of ZnO NPs synthesized from piperine against all the tested isolates remained unchanged after being exposed to proteinase- K. Nevertheless, ZnO NPs treated with enzymes exhibited a two- to four-fold increase in the MBC value against all the tested isolates (Supplementary Table 6). The green synthesized ZnO NPs were found to be variably stable to protease enzymes, as their antimicrobial activity evidenced by MIC and MBC values remained more or less similar, despite strain-wise variations being observed. This could be due to the destabilization of the bacterial cell membrane in the presence of protease enzymes [57]. Besides, the piperidine ring in piperine plays a crucial role in stabilizing ZnO NPs. Its steric-electronic properties, arising from interactions among the aromatic ring, carbonyl group, and alkene system, enhance the capping and protective effects of piperine on the NPs. This stabilization helps maintain the structural integrity of ZnO NPs, even in the presence of protease enzymes, by preventing enzymatic degradation or agglomeration [56]. Further, the protease stability of ZnO NPs could be improved by providing an energy barrier that might induce repulsion between these particles. Additionally, non-ionic macromolecules like polyethylene glycol (PEG) polymers and mucoadhesive polymers like polyacrylate can be used to reduce the interaction between NPs and inhibitors present in biological fluids and to limit the activity of endopeptidases [57].

#### Effect of physiological concentration of cationic salts

The stability of physiological cationic salts in the biological system is another barrier to deeming ZnO NPs fit as a suitable therapeutic candidate. These cationic salts can drastically change the colloidal stability and antibacterial capabilities once they enter the biological system [58]. Therefore, this study evaluated the stability of green synthesized ZnO NPs at physiological concentrations of cationic salts (150 mM NaCl and 2 mM MgCl_2_).

The green synthesized ZnO NPs from piperine maintained their antibacterial activity (MIC and MBC values) throughout the incubation period regardless of the cationic salts (150 mM NaCl and 2 mM MgCl_2_) (Supplementary Table 8). This stability may result from the functional groups and capping characteristics of the extracts utilized in the green synthesis of ZnO NPs [59].

#### Effect of pH

The intrinsic features of NPs, such as their stability, size, zeta potential, and morphology, can be impacted by the changes in the physicochemical parameters, such as pH and ionic strength of the solution [60]. Additionally, the pH significantly influences the stability and aggregation of NPs as it can change the interactions between NPs [61]. Therefore, the stability of green synthesized ZnO NPs at different pH levels (4.0, 6.0, and 8.0) was ascertained by evaluating their antibacterial activity against the MDR strains of *S*. Typhimurium and *S*. Enteritidis.

The MIC and MBC values of the ZnO NPs at pH 4.0, and 6.0 increased two-fold. Moreover, to our surprise, the antimicrobial activity of green synthesized ZnO NPs was reduced to half at pH 8.0, as evidenced by their MIC and MBC values (Supplementary Table 9). It is hypothesized that a low pH (acidic) would cause NPs to aggregate, decreasing their antibacterial activity. In contrast, a rise in the pH (alkalinity) of the solution would enhance the electrostatic repulsive force between NPs, increasing their antibacterial activity [62].

### In vitro safety assays

#### Haemolytic assay

Another barrier to the clinical application of ZnO NPs is their safety in operating only on pathogenic bacteria and not on mammalian cells. The in vitro haemolytic assay is often employed as a versatile assay for screening the toxicity of a therapeutic compound [63].

The present study observed dose-dependent hemolysis [64]. In addition, the green synthesized ZnO NPs exhibited minimal hemolysis (less than 2%) at the varied concentrations (1X; 5X; and 10X MIC), suggesting its safety on chicken erythrocytes. Moreover, it is suggested that when compared to bacterial cells, ZnO NPs at lower concentrations cannot produce toxicity in the biological system [65].

#### MTT cytotoxicity assay

Although the in vitro haemolytic assay can be successfully used to evaluate the preliminary toxicity studies, further cytotoxicity assays would provide a strong foundation for the results of the haemolytic studies and would further aid in assessing a drug candidate.

The MTT assay was used to evaluate the in vitro cytotoxicity effect of green synthesized ZnO NPs on the viability of HEK cell lines. Overall, the green synthesized ZnO NPs when treated at different concentrations (1X, 5X, and 10X MIC values) exhibited no cytopathic effect. Capping agents on the surface of green synthesized ZnO NPs may be responsible for the minimal cytotoxicity [64]. Furthermore, the low cytotoxicity exhibited by ZnO NPs might be due to the collateral sensitivity induced by piperine to selectively act on the bacterial cells [66].

#### Effect of biofabricated ZnO NPs on commensal gut lactobacilli

**Fig. 3:**
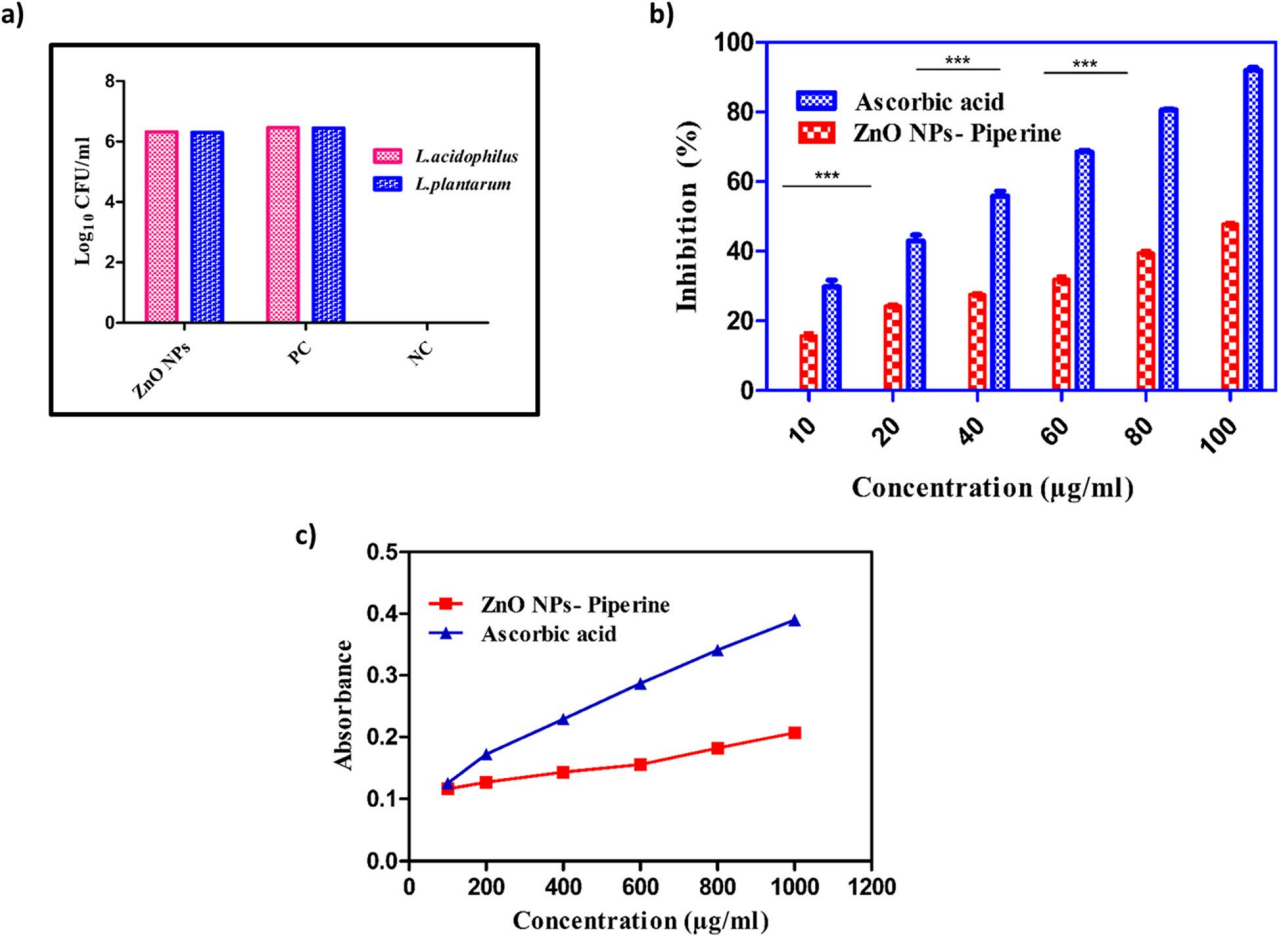
In vitro safety and antioxidant assay of ZnO NPs. Effect of ZnO NPs synthesized from piperine on commensal gut lactobacilli (**a**), ABTS assay (**b**), and reducing power assay (**c**)

As a crucial component of the body’s innate defense mechanism, it is equally necessary to examine how the ZnO NPs affect the commensal gut lactobacilli [67]. The beneficial gut lactobacilli revealed a similar growth pattern at 1X MIC on treatment with ZnO NPs synthesized using piperine (Fig. 3a). Overall, a non-significant (*P* > 0.05) antimicrobial effect was observed for the green synthesized ZnO NPs tested against *L. acidophilus* and *L. plantarum*. Hence, this study suggested that the ZnO NPs were safe for beneficial gut lactobacilli. Additionally, it is proposed that piperine is safe against the tested cultures of various microbiota at concentrations ≥ 0.10 mg mL^−1^, and piperine together with curcumin exhibited an average increase of 69% in the species identified within the gut microbiota [68].

### Antioxidant activity assay

Antioxidants play a crucial role in the functioning of all biological systems due to their significance in scavenging the toxic free radicals generated in the body, thereby preventing oxidative stress [69]. Hence, the antioxidant scavenging activity of the green synthesized ZnO NPs was assessed by employing ABTS and reducing power assay, keeping ascorbic acid as the standard.

In this study, ABTS and reducing power assays of the ZnO NPs revealed a dose-dependent increase in their antioxidant properties, indicating an improved ability to scavenge free radicals (Fig. 3b). The ZnO NPs could function as electron donors and interact with free radicals to terminate free radical chain reactions [50]. Nevertheless, the antioxidant activity of the ZnO NPs was lower when compared to the standard antioxidant, ascorbic acid [70]. The ZnO NPs exhibited better antioxidant activity than their bulk materials owing to their high surface area to volume ratio [71]. In addition, piperine has been reported to exhibit excellent antioxidant properties that prevent oxidative degradation in the biological system [70]. Moreover, experimental evidence from in vitro studies has demonstrated the protective effect of piperine against oxidative damage by quenching or inhibiting free radicals, hydroxyl radicals, and reactive oxygen species [71].

### Antibiofilm potential of ZnO NPs

The growing danger posed by biofilms to various fields of biomedicine is a global concern. Pathogenic bacteria use the formation of biofilm communities as a key strategy to enter host cells and promote the spread of infection [72]. Numerous issues arise from the ineffective management of microbial biofilms, including biodeterioration, food safety, and public health concerns [73]. ZnO NPs could be a viable strategy for treating bacterial biofilms due to their potential as an antibacterial agent. In this study, we investigated the ability of green synthesized ZnO NPs to inhibit the biofilm-forming ability of MDR-NTS isolates over 24 and 48 h employing CV- staining assay.

**Fig. 4:**
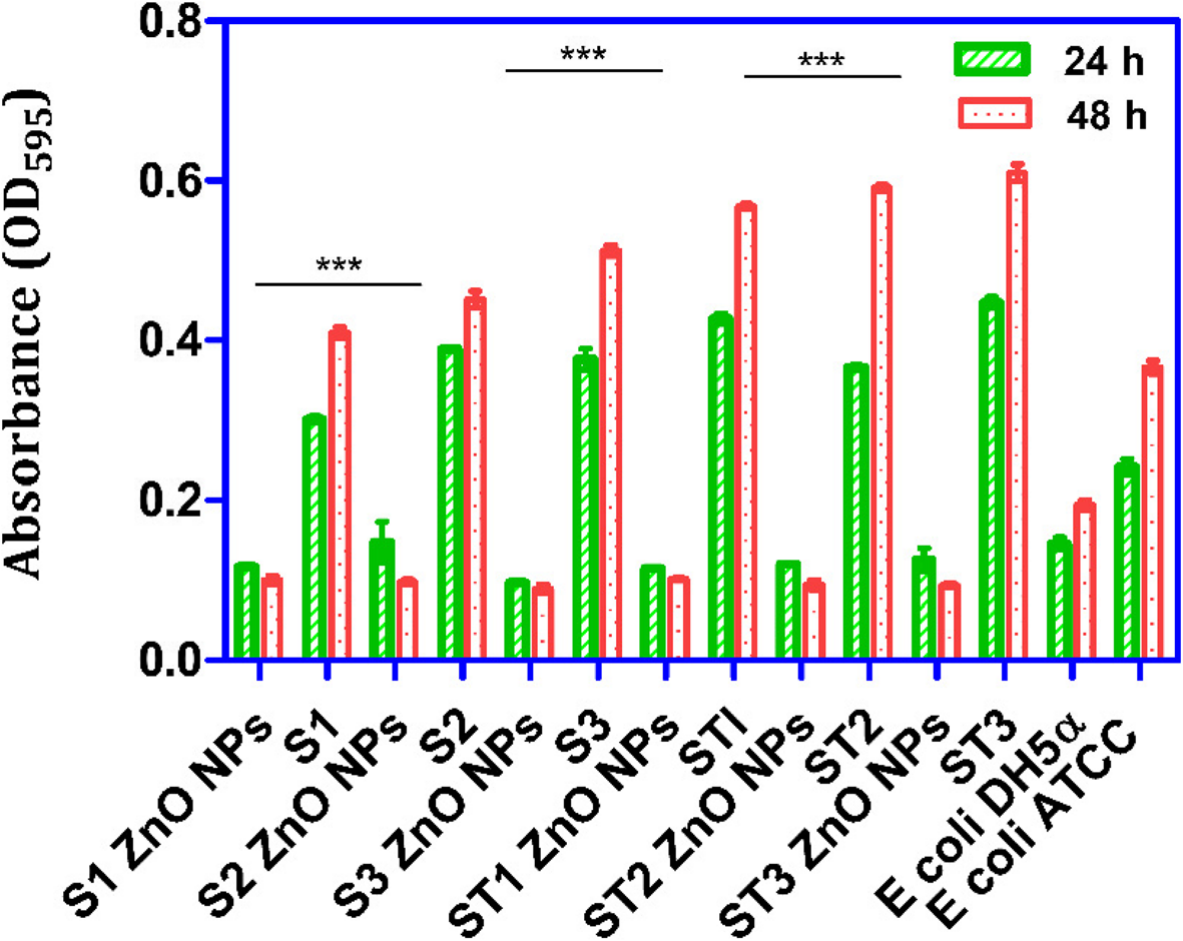
Inhibition of MDR-NTS biofilm by ZnO NPs using CV staining. Inhibition of MDR-NTS biofilm at 24 h and 48 h when treated with green synthesized ZnO NPs piperine using CV staining. Error bars indicate the standard deviation between strains. Positive and negative control bars indicate corresponding untreated MDR-NTS strains and *E. coli* DH5α biofilms, respectively (***P < 0.001; ** P < 0.01)

By using CV staining, it was determined that the ZnO NPs at 1X MIC exhibited a highly significant antibiofilm effect after 24 h (*P* < 0.001) because all the tested MDR-NTS isolates revealed a decrease in biomass, relative to their respective controls (untreated bacterial cultures). In addition, all the MDR-NTS isolates treated with the ZnO NPs after 48 h exhibited a significant inhibition (*P* < 0.001) of biofilm-forming ability (Fig. 4). Furthermore, it was observed that the biofabricated ZnO NPs could actively reduce the biofilm formation of the MDR-NTS isolates more effectively at 48 h as compared to 24 h (Supplementary Table 10).

The antibiofilm activity exhibited in this study demonstrated that the ZnO NPs may effectively inhibit the development of biofilm by influencing the proliferation of pathogenic bacteria. This inhibition of biofilm formation could be due to the reduction in the adhesion of bacterial cells by the green synthesized ZnO NPs to the matrix provided, thereby interfering with the formation of bacterial biofilms [74, 75]. Since the MDR-NTS biofilms were susceptible to the ZnO NPs, they can be considered an excellent candidate for preventing and inhibiting biofilm formation by bacterial pathogens.

### In vitro killing kinetics of MDR-NTS strains treated with ZnO NPs

**Fig. 5:**
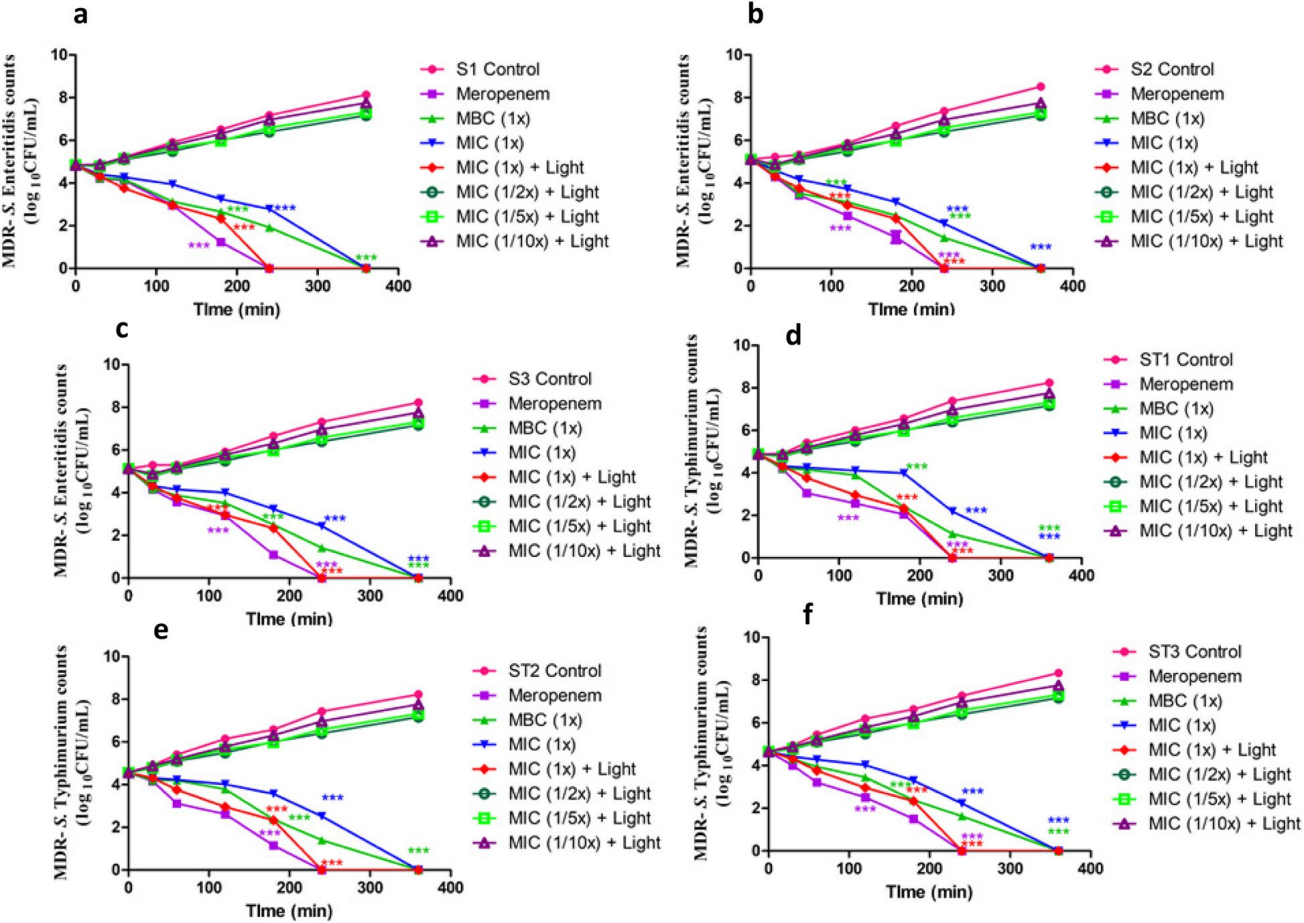
In vitro dose- and time-dependent extracellular killing kinetics of MDR-NTS strains treated with ZnO NPs from piperine. Three MDR isolates each of *S*. Enteritidis (**a**, **b**, **c**) and *S*. Typhimurium (**d**, **e**, **f**) were co-cultured with MBC and MIC values of green synthesized ZnO NPs from piperine in CA-MH broth at 37 °C under static conditions. Simultaneously, respective controls (untreated and meropenem-treated) of MDR isolates were incubated in CA-MH broth. The photocatalytic disinfection is estimated by exposing the bacterial culture treated with ZnO NPs on exposure to LED light at their MIC and sub-MIC levels. The data expressed as log_10_CFU/mL represents the mean ± standard deviation of three independent experiments. Error bars are too close to display. (***P < 0.001)

By investigating the in vitro extracellular dose- and time-dependent kinetics assay of MDR-NTS strains, the antibacterial pattern of ZnO NPs was evaluated. The untreated MDR-NTS strains, in this study, exhibited an increasing growth pattern over time. However, the complete elimination of MDR-NTS strains was observed after 4 h in all the bacterial strains treated with meropenem as antibiotic control. Interestingly, no difference was observed in the time-kill assay when the tested MDR- NTS strains were treated with MIC and MBC levels of ZnO NPs (Fig. 5). The antibacterial activity exhibited by the ZnO NPs and meropenem might be due to the peculiar cell wall structure of Gram-negative bacteria which possess a thin peptidoglycan layer and an outer membrane composed of lipopolysaccharides [72]. The results of the time-kill assay suggested that the ZnO NPs synthesized from piperine possess a broad spectrum of antibacterial activity, as they exert a similar effect on all the tested MDR strains of *Salmonella* spp.

### In vitro photocatalytic disinfection of MDR-NTS strains treated with ZnO NPs

The photocatalytic growth kinetics of individual MDR isolates of *S*. Typhimurium and *S*. Enteritidis co-cultured with different concentrations (1X, 1/2X, 1/5X, and 1/10X MIC) of biofabricated ZnO NPs along with their respective controls was performed on exposure to LED light (Fig. 5). This study did not employ the use of either sunlight or UV light because they have proven bactericidal activity [76]. The MDR isolates of *S*. Typhimurium and *S*. Enteritidis exhibited a progressively increasing growth pattern at 30, 60, 120, 180, and 240 min of incubation (Fig. 5). In this study, the MDR bacterial test strains when treated with ZnO NPs (1X MIC) exhibited a progressive decline in bacterial growth after 30 min of co-incubation. Surprisingly, none of the MDR test strains (*S*. Typhimurium and *S*. Enteritidis) exhibited visible growth after 240 min of co-incubation (*P* < 0.001) with ZnO NPs at 1X MIC (Fig. 5). Regardless of the MDR-test strains, no bacterial elimination could be observed when treated with sub-MIC levels (1/2X, 1/5X and 1/10X MIC) of ZnO NPs (Fig. 5). The pronounced antibacterial activity exhibited at a much lesser time when compared to the non-photocatalytic bacterial elimination could be probably due to the higher quanta of ROS generation from the ZnO NPs which actively participated in bacterial killing upon photoexcitation [77].

### Photocatalytic dye degradation abilities of biofabricated ZnO NPs

**Fig. 6:**
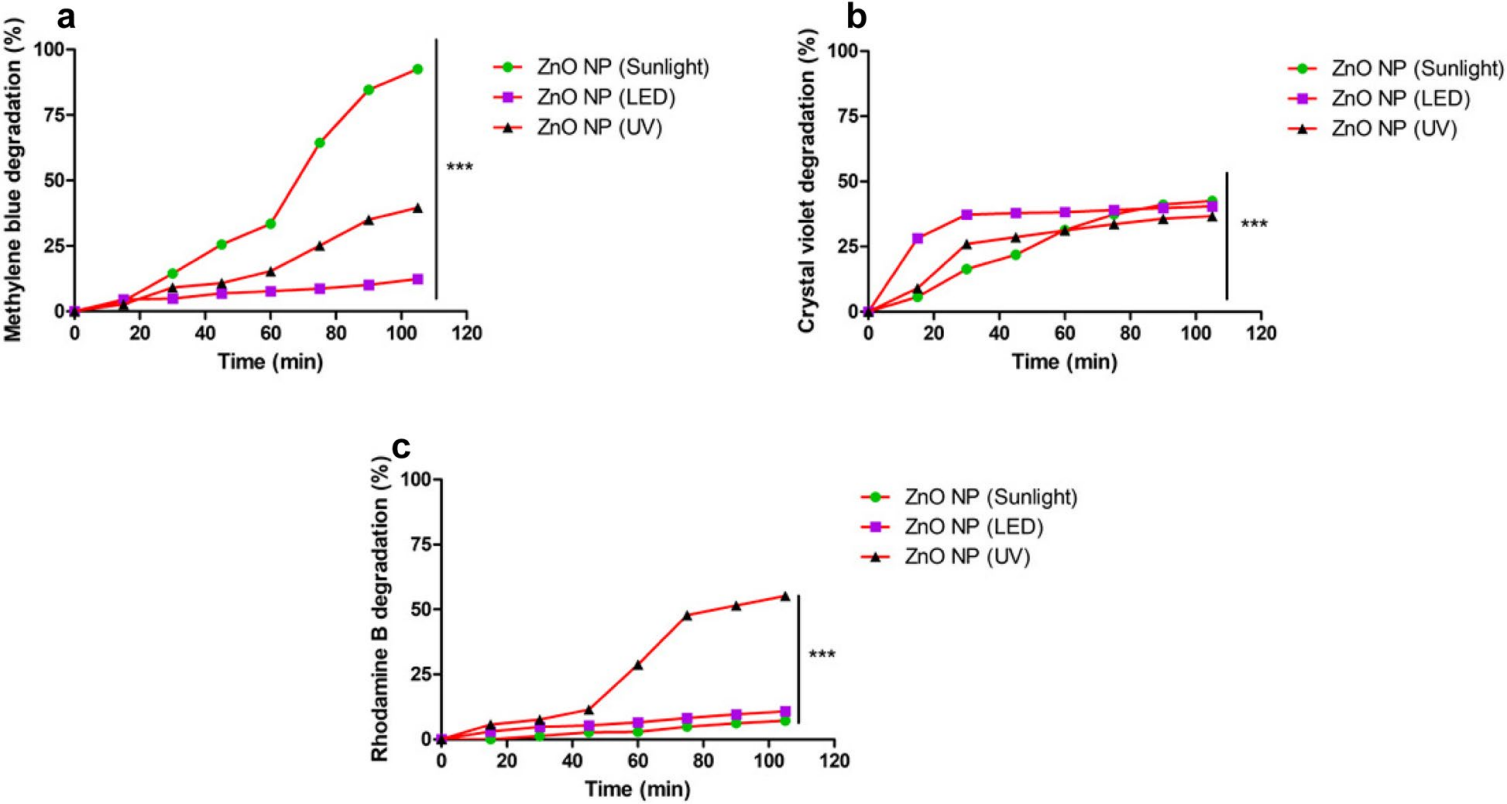
Photocatalytic dye degradation ability of ZnO NPs. Images represent the degradation ability of the ZnO NPs when treated with Methylene blue (**a**), Crystal violet (**b**), and Rhodamine-B (**c**) on exposure to three different sources of light sources viz., sunlight, LED light and UV light

Regardless of the light source, ZnO NPs exhibited a gradual increase in the photodegradation of all tested dyes (MB, CV, and RhB) over time. The dye degradation potential of the ZnO NPs in the dark was negligible compared to the photocatalytic degradation under light. On exposure to sunlight for 105 min, MB displayed a progressive increase in degradation up to 92.53% and CV exhibited 42.51%, whereas RhB exhibited a minimal degradation of 7.22% (Table 1; Fig. 6; Supplementary Fig. 3).

**Table 1:** Determination of photocatalytic degradation potential of dyes on treatment with ZnO NPs under different light sources

	**Sunlight**			**LED light**			**UV light**		
	**MB**	**CV**	**RhB**	**MB**	**CV**	**RhB**	**MB**	**CV**	**RhB**
**Time (min)**	**Degradation (%)**								
**15**	3.94	5.71	0	4.56	28.23	3.01	2.73	9.02	5.71
**30**	14.46	16.35	1.33	4.86	37.27	4.76	9.11	26.07	7.61
**45**	25.51	21.84	2.67	6.91	37.88	5.41	10.77	28.66	11.45
**60**	33.44	31.36	2.86	7.67	38.21	6.52	15.35	31.21	28.83
**75**	64.40	37.39	4.88	8.67	39.07	8.19	25.15	33.59	47.82
**90**	84.62	41.14	6.23	10.14	39.79	9.62	35.05	35.75	51.50
**105**	92.53	42.51	7.22	11.56	40.48	10.80	42.32	39.33	56.13
**Kinetics**									
**Order**	Second	Second	Zero	Second	Second	Zero	Zero	Second	Zero
**R**^**2**^** value**	0.944	0.960	0.970	0.944	0.631	0.974	0.943	0.924	0.909
**Intercept**	0.001	0.007	0.000	0.001	0.002	−0.001	−0.003	0.003	−0.007
**Slope**	0.939	0.861	1.185	0.939	0.657	1.127	1.005	0.551	1.225

Sunlight was chosen as a light source due to its natural availability over a large area with a diverse spectrum of wavelengths in nearly equal proportions. Sunlight contains around 45% visible spectra and 5% UV irradiation [78]. LEDs were also selected owing to their low energy usage, easy availability, and long shelf life. UV light was also used as it consists of high-frequency, non-ionizing radiation with short wavelengths from 100–300 nm. This produces high-energy photons due to the high photoexcitation potential on photocatalyst surfaces. In this study, the ZnO NPs performed well with UV light over LED (Table 1; Supplementary Fig. 3) for the reason that ZnO NPs expend their photocatalytic potential due to the formation of excitons which maximizes at 340–380 nm and thus exhibit better photocatalysis [79]. The photocatalytic dye degradation mechanisms involve dye sensitization through charge induction, indirect dye oxidation/reduction reactions, and direct photolysis of dye molecules [80]. Photodegradation occurs mainly through oxidation by positive valence band holes, reduction by conduction band electrons, and attack by hydroxyl radicals. When a nanocatalyst is exposed to light, electron–hole pairs are generated on its surface, driving photocatalytic degradation. Reactive dyes interact with hydroxyl groups to form hydroxyl radicals or are oxidized by the holes, leading to less toxic breakdown products [81]. Upon activation by UV light, ZnO NPs initiate a redox reaction, producing free radicals like OH and O₂, which decompose the dye into smaller organic molecules [82]. Furthermore, the stability of RhB towards degradation could be attributed to its high stability and solubility in water [83], and the variation in their photocatalytic behaviour may be due to differences in their band gaps owing to the much higher energy required for the photoexcitation of ZnO NPs.

The reaction parameters obtained showed that RhB was fitted in zero-order kinetics irrespective of the light sources. Besides, MB followed primarily second-order kinetics except for the reaction with exposure to UV light, wherein it followed zero-order kinetics. Similarly, CV mainly followed second-order kinetics throughout the experiment (Table 1). The variation in the order of reaction for dye degradation by ZnO NPs under different light sources may be attributed to the generation of hot charge carriers, increased temperatures, and focused electromagnetic fields upon absorption and scattering of light from the various sources [84].

In short, this study explores an eco-friendly method for synthesizing ZnO NPs using piperine, a potential antibacterial compound. The synthesized ZnO NPs exhibited significant antibacterial activity against MDR strains of *Salmonella* spp., suggesting potential clinical applications as alternatives to traditional antibiotics. Furthermore, their ability to disrupt biofilms enhances their therapeutic potential, particularly in chronic infections where biofilms contribute to antibiotic resistance. Environmentally, ZnO NPs exhibited effective photocatalytic properties, degrading harmful dyes such as MB, CV, and RhB under visible light, thus offering a promising approach for wastewater treatment. The degraded products could potentially be less toxic than their parent compounds; however, thorough ecotoxicological assessments are necessary to ensure they do not pose environmental or health risks. It is essential to evaluate the toxicity of these byproducts on aquatic life and other environmental components to ensure that the photocatalytic degradation process does not introduce new hazards. Furthermore, scalability challenges in the synthesis process can be addressed by optimizing synthesis parameters, standardizing the source and purity of piperine, adopting cost-effective production methods, and implementing automated synthesis systems. By improving these aspects, the eco-friendly synthesis of ZnO NPs can be made more viable for industrial applications, enhancing both their clinical and environmental utility [85, 86].

## Conclusions

In this study, we investigated an eco-friendly approach for synthesizing ZnO NPs using piperine. Piperine was observed to be a potential antibacterial compound with minimal toxicity and was found to interact strongly with the ompC motif of *Salmonella* spp. by in silico ADMET analysis and molecular docking. The colour of the reaction solution changed, indicating the synthesis of ZnO NPs, which corroborated with a maximum absorbance peak at 340 nm in UV–Vis spectroscopy. Meanwhile, the PXRD and TEM analysis of ZnO NPs revealed a hexagonal wurtzite crystalline structure. The synthesized ZnO NPs exhibited MIC and MBC levels of 62.50 µg/mL and 125 µg/mL, respectively. In addition, the ZnO NPs synthesized from piperine appeared to be variably stable and safe against the tested MDR-NTS strains with minimal hemolysis against chicken RBCs and no cytopathic effect against HEK cell lines. Further, the ZnO NPs exhibited significant antibiofilm activity against the tested MDR-NTS strains as well as a concentration-dependent antioxidant activity. The antimicrobial effect coupled with minimal hemolysis suggests that ZnO NPs could be a viable therapeutic alternative to traditional antibiotics, which are increasingly ineffective against MDR pathogens. By disrupting biofilm integrity, ZnO NPs could enhance the effectiveness of existing treatments, especially in chronic or persistent infections where biofilms often render pathogens resistant to conventional antibiotics. Moreover, the ZnO NPs exhibited photocatalytic disinfection and complete elimination of MDR-NTS strains at 240 min in comparison to 360 min without exposure to LED light. Furthermore, ZnO NPs significantly degraded the tested cationic dyes (MB, CV, and RhB) when exposed to sunlight, UV, and LED light. Consequently, the analysis demonstrated an eco-friendly means to synthesize ZnO NPs from piperine, which might be used as a viable antibacterial candidate with antioxidant, antibiofilm as well as photocatalytic properties. The unique structural configurations of the ZnO NPs have also opened further ways to use them as a photocatalyst, exhibiting efficient disinfection and dye degradation abilities under visible light sources for devising wastewater treatment. Further strategies need to be formulated to improve the antimicrobial efficacy as well as the photocatalytic potential of the green synthesized ZnO NPs, either by doping with other NPs or by optimizing different conditions employed for the synthesis so that the material could be used for commercial wastewater treatment.

The piperine-mediated synthesis of ZnO NPs exhibited notable antibacterial and photocatalytic properties; however, scalability for industrial applications poses challenges due to the need for stringent control of synthesis parameters, such as temperature and pH. Furthermore, the bioactivity of piperine may vary depending on its source and purity, potentially affecting the reproducibility of results. Moreover, biocompatibility assessments are essential to ensure the safe use of these nanoparticles, particularly for applications involving living systems. To enhance the practical applicability of this method, it is crucial to address these challenges while also evaluating cost-efficient production methods and the environmental implications of large-scale nanoparticle synthesis. Additionally, there is a need to develop a reliable and standardized method for producing NPs with consistent morphology, size, and homogeneity to ensure their practical applications. Hence, future research should aim to further optimize the synthesis parameters, regeneration efficacy, the structural integrity of ZnO NPs after repeated cycles, and the impact of piperine on maintaining their photocatalytic performance in vitro as well as in diverse host environments. In addition, in vivo clinical studies on suitable host models are essential to thoroughly evaluate the clinical efficacy and safety profiles.

## Supplementary Information

Supplementary Material 1.

## Data Availability

All data generated or analysed during this study are included in this published article (and its supplementary information files). The datasets used and/or analysed during the current study are available from the corresponding author on reasonable request.
