# Supplementary material for: Antibacterial and photocatalytic potential of piperine-derived zinc oxide nanoparticles against multi-drug-resistant non-typhoidal Salmonella spp
Source: BMC Microbiol. 2025 Feb 25;25:89. doi: 10.1186/s12866-025-03829-4 (PMC11852875; doi:10.1186/s12866-025-03829-4)
Supplement: Supplementary file 1 — Supplementary Material 1. [file 12866_2025_3829_MOESM1_ESM.docx]

**Elucidating antibacterial and photocatalytic potential of zinc oxide nanoparticles synthesized from piperine against multi-drug-resistant non-typhoidal *Salmonella* spp.**

Varsha Unni^a#^, Bibin Mohan^a#^, Padikkamannil Abishad^a^, Pokkittath Radhakrishnan Arya^a^, Sanis Juliet^b^, Lijo John^c^, Valil Kunjukunju Vinod^a^, Asha Karthikeyan^a^, Nitin Vasantrao Kurkure^d^, Sukhadeo Baliram Barbuddhe^e^, Deepak Bhiwa Rawool^e^* and Jess Vergis^a^*

^#^Equal contribution

^a^Department of Veterinary Public Health, College of Veterinary and Animal Sciences, Pookode, Kerala Veterinary and Animal Sciences University, Wayanad- 673 576, India

^b^Department of Veterinary Pharmacology and Toxicology, College of Veterinary and Animal Sciences, Pookode, Kerala Veterinary and Animal Sciences University, Wayanad- 673 576, India

^c^Department of Veterinary Biochemistry, College of Veterinary and Animal Sciences, Pookode, Kerala Veterinary and Animal Sciences University, Wayanad- 673 576, India

^d^Department of Veterinary Pathology, Nagpur Veterinary College, Nagpur- 440 006, India

^e^ICAR- National Meat Research Institute, Hyderabad- 500 092, India

***Corresponding authors:**

**J Vergis**: College of Veterinary and Animal Sciences, Pookode, KVASU, Wayanad- 673 576, India; **Tel**: +91-9446355683; **Email**: [itzjessvergis@gmail.com](mailto:itzjessvergis@gmail.com)

**D.B. Rawool**: ICAR-National Meat Research Institute, Hyderabad- 500 092, India; **Tel**: +91-9412672852; **Email**: [deepak.rawool@yahoo.com](mailto:deepak.rawool@yahoo.com)

**S1. *In silico* molecular docking of piperine**

The protonated low-energy 3-D conformation of piperine was constructed using Chem 3D v.16.0. A blind docking employing Autodock v.4.20 was carried out to assess the binding affinity of piperine to ompC motifs of *Salmonella* spp. By eliminating the ligand, the water molecule, the hetero atoms, and the co-crystallized solvents, the target proteins were generated. Polar-charged hydrogen was added along with Gasteiger charges, and non-polar hydrogens were combined. Besides, a grid map with 60x60x60 points and a 0.375 Å spacing was produced. The docking probability was further examined using the Lamarckian genetic algorithm. The ten best poses generated by the configuration files for OmpC motifs with piperine were recorded using the software. The ligand was scored according to docked energy, and molecular docking was shown using a Pymol viewer.

**S2. Effect of high-end temperatures**

In order to determine the thermal stability of green synthesized ZnO NPs using piperine, they were subjected to high-end temperatures (70 ^o^C and 90 ^o^C) for 5, 15, and 30 min. Subsequently, the antimicrobial activity (MIC and MBC values) of the green synthesized ZnO NPs was determined against each of the MDR- NTS test strains, corresponding to each time interval. The untreated ZnO NPs, kept at room temperature, were used as a control for each time interval (Vergis et al., 2021).

**S3. Effect of protease enzymes (trypsin, lysozyme, and proteinase-K)**

The effect of protease enzymes (trypsin, lysozyme, and proteinase- K) on the antimicrobial activity of green synthesized ZnO NPs using piperine was investigated by co-incubating with the respective proteases at 37 ^o^C for 30 sec, 5, 15, and 30 min (Vergis *et al*., 2021).

The protease to ZnO NP ratio used in this assay was 1:100 (w/w). Each of the protease- untreated ZnO NP was used as a control to rule out the antimicrobial activity. After incubation at respective time intervals, the samples were heated at 90 ^o^C for 10 min in order to inactivate the protease activity and then the antimicrobial activity (MIC and MBC values) was determined.

**S4.** **Effect of physiological concentration of cationic salts**

In order to investigate the stability of green synthesised ZnO NPs using piperine, the presence of a physiological concentration of cationic salts, the NPs were tested against MDR- NTS isolates (*S*. Typhimurium and *S*. Enteritidis; n= 3 for each serotype) in a CA-MH broth with added concentrations of NaCl (150 mM). Subsequently, the MIC and MBC values were identified (Vergis et al., 2021).

**S5. Effect of pH**

In order to investigate the stability of green synthesised ZnO NPs using piperine at different pH, the NPs were tested against the individual serotypes of both *S*. Typhimurium and *S*. Enteritidis in CA-MH broth maintained at pH four, six and eight. Subsequently, the MIC and MBC values were identified (Vergis et al., 2021).

**S6. Haemolytic Assay**

In order to determine the safety of green synthesised ZnO NPs using piperine (to ensure their activity specifically against the MDR-NTS isolates and not the mammalian cells), the haemolytic assay based on the release of haemoglobin from chicken erythrocytes was performed in polystyrene microtitre plates (Vergis et al., 2021).

In brief, the aseptically collected fresh defibrinated chicken blood was washed thrice with PBS, centrifuged for 15 min at 1000 xg and resuspended at 10% (v/v) in PBS containing 10 mM DL-Dithiothreitol (DL- DTT). The chicken erythrocytes (100 μL) were then transferred to a 96- well microtiter plate and mixed with 100 μL of ZnO NP solution kept at different MIC (1X, 5X and 10X) levels. Sterile PBS (100 μL) was used as a negative control, whereas 0.20 per cent Triton X-100 (100 μL) was used as the positive control. The microtiter plates were then incubated at 37°C for 60 min and further centrifuged at 1300 xg for 10 min. The supernatant was transferred to a flat-bottom 96-well polystyrene microtiter plate and the haemoglobin release was monitored by measuring the absorbance at 540 nm. The percentage of haemolysis was calculated as,

**Haemolysis = 100 x (A_sample_ − A_PBS_)/ (A_TritonX-100_ – A_PBS_)**

wherein, A_sample_ is the experimental absorbance of the NP sample, A_PBS_ is the control absorbance of untreated erythrocytes and A_TritonX-100_ is the absorbance of 0.20 per cent Triton X-100 lysed cells.

**S7. MTT cytotoxicity assay**

The *in vitro* effect of green synthesised ZnO NPs using piperine on the viability of eukaryotic cells was evaluated using MTT assay [3-(4,5-dimethylthiazole-2-yl)-2,5-diphenyl tetrazolium bromide] (Vergis et al., 2021). In this study, the viability of ZnO NPs was tested against eukaryotic HEK cell lines.

In brief, the HEK cells were pre-cultured in tissue culture flasks containing Dulbecco’s Modified Eagle Medium (DMEM, pH 7.20) until the formation of a monolayer at the bottom of the flasks. The adherent HEK cells were then transferred to another 96-well plate at a density of 1x10^5^ cells per well and allowed to attach overnight. The monolayers of cells were treated with 200 µL of ZnO NPs at different MIC (1X, 5X and 10X) levels diluted in DMEM; the treated cells were maintained for 24 h at 37 °C in a humidified incubator with 5% CO_2_ atmosphere. The cells incubated with fresh DMEM served as the negative control. The supernatant was then removed and proceeded further using the MTT cell proliferation assay kit (Abcam, USA). Accordingly, 50 µL of MTT reagent mixed with 50 µL of media was added to each well and incubated at 37 °C for three h. After incubation, MTT solvent (150 µL) was added to each well, mixed thoroughly and the cytotoxicity was monitored by measuring the absorbance at 590 nm. The percentage of cytotoxicity was calculated as,

**Cytotoxicity = 100 x (Control– Sample)/ (Control)**

wherein, the control denotes experimental absorbance of the untreated cell control and the sample represents the control absorbance of the treated cell lines.

**S8. Effect of ZnO NPs on commensal gut lactobacilli**

The adverse effect of green synthesised ZnO NPs using piperine was further explored on commensal gut lactobacilli (*L.* *acidophilus* MTCC 10307 and *L. plantarum* MTCC 5690).

In brief, de Mann- Rogosa- Sharpe (MRS) broth medium (100 µL) containing 1X MIC levels of ZnO NPs was inoculated with a defined number of each commensal bacteria (1x10^7^ CFU/mL) in 96-well microtiter plates. Each plate included a positive growth control (untreated individual commensal lactobacilli) and negative control (sterile MRS broth). After incubation at 37 °C for 48 h, the effect of ZnO NPs on commensal lactobacilli was determined by drawing two samples (10 µL) from each well and plated onto MRS agar plates (Vergis et al., 2021).

**S9. Reducing power assay**

In the presence of antioxidants, Fe^3+^ gets reduced to Fe^2+^ which serves as the basis for the reducing power assay. In brief, sodium phosphate buffer (0.20 M, 0.50 mL; pH 6.60) and potassium ferricyanide (30 mM, 0.50 mL) were added to 1 mL of biofabricated ZnO NPs at varying concentrations (100, 200, 400, 600, 800, 1000 µg/mL), keeping ascorbic acid as control. After incubation at 50 ^o^C for 20 min, the mixture was centrifuged (5000 rpm; 10 min), followed by the addition of trichloroacetic acid (TCA, 0.60 M, 2 mL) and centrifugation (3000 rpm; 10 min). The supernatant obtained after centrifugation (0.50 mL) was mixed with 0.10 mL of FeCl_3_ solution (6 mM) and deionized water (0.50 mL). Further, the optical density at 700 nm was measured.

**S10. ABTS assay**

The antioxidant activity of biofabricated ZnO NPs was evaluated using the 2,2′- azinobis (3-ethylbenzothiazoline-6-sulfonic acid; ABTS) radical (Dalmolin et al. 2016). Initially, ABTS^•+^ radical was generated in the dark by mixing ABTS (7 mM) and potassium persulfate (140 mM) at room temperature for 15 h. The absorbance of ABTS^•+^ solution was adjusted to 0.70 by mixing with 10 mM phosphate buffer (pH 7.40) at 734 nm. Meanwhile, the biofabricated ZnO NPs were prepared at varying concentrations (10, 20, 40, 60, 80, and 100 µg/mL); ascorbic acid serves as the standard. Further, the absorbance of treated samples was read at 734 nm after its incubation (37°C for 20 min) in the dark, and the free-radical scavenging property was determined as,

$$\boldsymbol{Free radical scavenging property (\%)=}\frac{\boldsymbol{A}_{\boldsymbol{Control}}\boldsymbol{-}\boldsymbol{A}_{\boldsymbol{Test}}}{\boldsymbol{A}_{\boldsymbol{Control}}}\boldsymbol{\times100}$$

wherein A_Control_ denotes the absorbance of the ascorbic acid, whereas A_Test_ denotes the absorbance of treated samples.

**S11. *In vitro* antibiofilm potential of biofabricated ZnO NPs**

Using the crystal violet staining assay in 96-well microtiter plates, the antibiofilm activity of biofabricated ZnO NPs was evaluated against the tested MDR-NTS strains both at 24 and 48 h (Vergis et al. 2019). In brief, MDR-NTS strains (10^7^ CFU/mL; 50 µL) and ZnO NPs (50 µL) were co-incubated in sterile nutrient broth supplemented with 0.45% D-Glucose (HiMedia) with suitable controls. The positive control consisted of respective untreated MDR-NTS strains (50 μL) in nutrient broth (50 μL), while the negative control consisted of sterile nutrient broth (100 μL). Besides, *E. coli* ATCC 25922 was used in this assay as a known biofilm-forming strain, and *E. coli* DH5α served as a non-biofilm former. The supernatant containing planktonic growth of MDR-NTS strains was removed from the microtitre plates after 24 h and 48 h of incubation at 37⁰C. Later, each well received 0.10% crystal violet stain (100 μL), which was kept for 30 min. The stain was then discarded, and the wells were rinsed using PBS (100 μL) three times; subsequently, stained biofilm formed at the bottom of each well was solubilized with 95% ethanol.

**S12. Photocatalytic degradation kinetics of cationic dyes treated with ZnO NPs**

To quantify the photocatalytic reaction kinetics, different kinetic models were analyzed as follows,

**Zero-order kinetics:** $\boldsymbol{C}_{\boldsymbol{1}}\boldsymbol{= -kt+}\boldsymbol{C}_{\boldsymbol{o}}$

**First-order kinetics:** $\boldsymbol{ln}\boldsymbol{C}_{\boldsymbol{1}}\boldsymbol{= -kt+ln}\boldsymbol{C}_{\boldsymbol{0}}$

**Pseudo-first order kinetics:** $\boldsymbol{ln}\frac{\boldsymbol{C}_{\boldsymbol{1}}}{\boldsymbol{C}_{\boldsymbol{0}}}\boldsymbol{= -kt}$

**Second-order kinetics:** $\frac{\boldsymbol{1}}{\boldsymbol{C}_{\boldsymbol{1}}}\boldsymbol{=kt+}\frac{\boldsymbol{1}}{\boldsymbol{C}_{\boldsymbol{0}}}$

wherein, the rate constants in the different order kinetics are expressed as Zero-order rate constant (k0): mol L^-1^ min^-1^; first-order rate constant (k1): min^-1^; pseudo-first-order rate constant (k1’): min^-1^; Second-order rate constant (k2): L mol^-1^ min^-1^.

**Supplementary Table 1: Antibiotic susceptibility testing of MDR-NTS isolates used in the present study**

| **ISOLATE ID** | | **GEN**  **(10mcg)** | **AMP**  **(2mcg)** | **C**  **(30mcg)** | **CL**  **(10 mcg)** | **AZM**  **(15mcg)** | **MRP**  **(10mcg)** | **DO**  **(30 mcg)** | **CIP**  **(5 mcg)** | **CTR**  **(30mcg)** | **COT**  **(25mcg)** | **AMC**  **(10mcg)** | **NA**  **(30 mcg)** | **ESBL** | |
| --- | --- | --- | --- | --- | --- | --- | --- | --- | --- | --- | --- | --- | --- | --- | --- |
|  |  |  |  |  |  |  |  |  |  |  |  |  |  | **CAZ (30 mcg)/CAC (30/10 mcg)** | **CTX (30 mcg)/CEC**  **(30/10 mcg)** |
| ***S*. Enteritidis** | S1 | **R** | **R** | **R** | S | I | S | **R** | **R** | S | **R** | **R** | **R** | S | S |
|  | S2 | **R** | **R** | **R** | S | I | S | **R** | **R** | S | **R** | **R** | **R** | S | S |
|  | S3 | **R** | **R** | S | S | I | S | **S** | **S** | S | **R** | **R** | **R** | S | S |
| ***v***  ***S*. Typhimurium** | ST1 | **R** | **R** | I | S | S | S | **R** | **R** | S | **R** | **R** | **R** | S | S |
|  | ST2 | **R** | **R** | **R** | S | I | S | **R** | **R** | I | **R** | **R** | **R** | **R** | S |
|  | ST3 | **R** | **R** | S | S | I | S | **R** | **R** | S | **R** | **R** | **R** | S | S |

(GEN- Gentamicin, AMP- Ampicillin, C- Colistin sulphate, CL- Chloramphenicol, AZM- Azithromycin, MRP- Meropenem, DO- Doxycycline, CIP- Ciprofloxacin, CTR- Ceftriaxone, COT- Co-trimoxazole, AMC: Amoxycillin, NA- Nalidixic acid, CAZ- Cefotaxime, CAC- Cefotaxime/ clavulanic acid, CTX- Ceftazidime, CEC- Ceftazidime/ clavulanic acid)

**Supplementary Table 2. *In silico* ADME analysis of piperine**

| **Sl. No.** | **Descriptors** | **Piperine** |
| --- | --- | --- |
| *Physico chemical properties* | | |
|  | Formula | C17H19NO3 |
|  | Molecular weight | 285.34 g/mol |
|  | Num. heavy atoms | 21 |
|  | Num. arom. heavy atoms | 6 |
|  | Fraction Csp3 | 0.35 |
|  | Num. rotatable bonds | 4 |
|  | Num. H-bond acceptors | 3 |
|  | Num. H-bond donors | 0 |
|  | Molar Refractivity | 85.47 |
|  | TPSA | 38.77 Å² |
| *Lipophilicity* | | |
|  | Log *P*_o/w_ (iLOGP) | 3.38 |
|  | Log *P*_o/w_ (XLOGP3) | 3.46 |
|  | Log *P*_o/w_ (WLOGP) | 2.51 |
|  | Log *P*_o/w_ (MLOGP) | 2.39 |
|  | Log *P*_o/w_ (SILICOS-IT) | 3.41 |
|  | Consensus Log *P*_o/w_ | 3.03 |
| Water Solubility | | |
|  | Log *S* (ESOL) | -3.74 |
|  | Solubility | 5.24e-02 mg/ml ; 1.84e-04 mol/l |
|  | Class | Soluble |
|  | Log *S* (Ali) | -3.96 |
|  | Solubility | 3.16e-02 mg/ml ; 1.11e-04 mol/l |
|  | Class | Soluble |
|  | Log *S* (SILICOS-IT) | -3.00 |
|  | Solubility | 2.87e-01 mg/ml ; 1.00e-03 mol/l |
|  | Class | Soluble |
| *Pharmacokinetics* | | |
|  | GI absorption | High |
|  | BBB permeant | Yes |
|  | P-gp substrate | No |
|  | CYP1A2 inhibitor | Yes |
|  | CYP2C19 inhibitor | Yes |
|  | CYP2C9 inhibitor | Yes |
|  | CYP2D6 inhibitor | No |
|  | CYP3A4 inhibitor | No |
|  | Log *K*_p_ (skin permeation) | -5.58 cm/s |
| *Drug-likeness* | | |
|  | Lipinski | Yes; 0 violation |
|  | Ghose | Yes |
|  | Veber | Yes |
|  | Egan | Yes |
|  | Muegge | Yes |
|  | Bioavailability Score | 0.55 |
| *Medical Chemistry* | | |
|  | PAINS | 0 alert |
|  | Brenk | 2 alerts: michael_acceptor_1, polyene |
|  | Lead likeness | Yes |
|  | Synthetic accessibility | 2.92 |

**Supplementary Table 3. *In silico* toxicity characteristics of piperine assessed by Protox-II and StopTox software**

| **Sl. No.** | **Endpoint** | **Prediction** | **Confidence (%)** | **Applicability domain (AD)** | **Predicted fragment contribution** |
| --- | --- | --- | --- | --- | --- |
| 1. | Acute inhalation toxicity | Non toxic (-) | 63 | 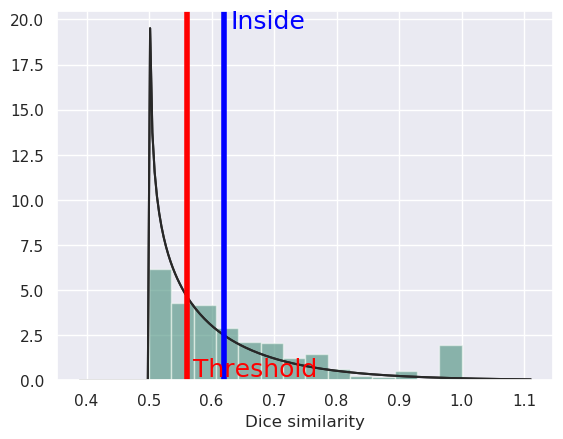 | 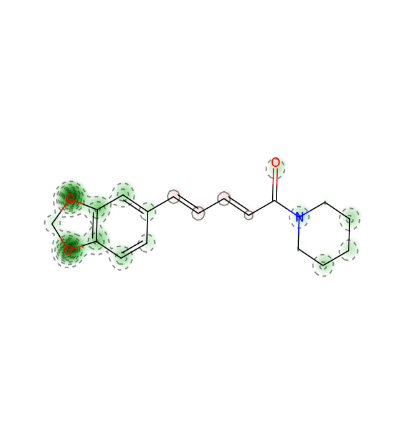 |
| 3. | Acute dermal toxicity | Non toxic (-) | 72 | 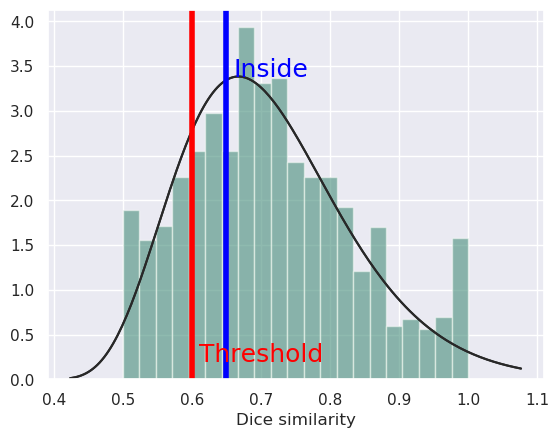 | 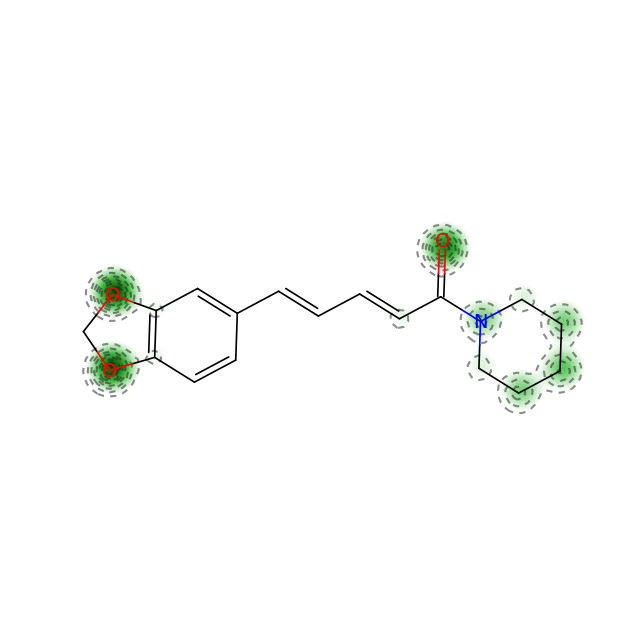 |
| 5. | Skin sensitization | Sensitizer | 60 | 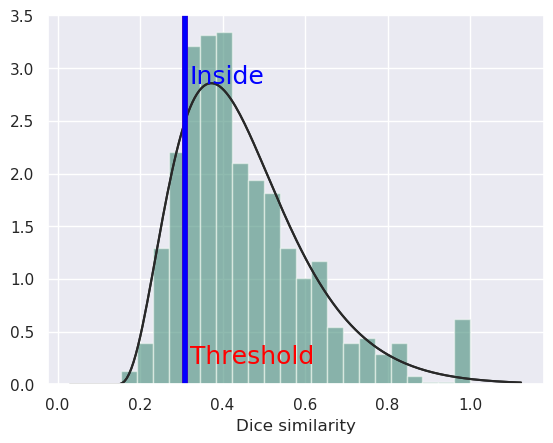 | 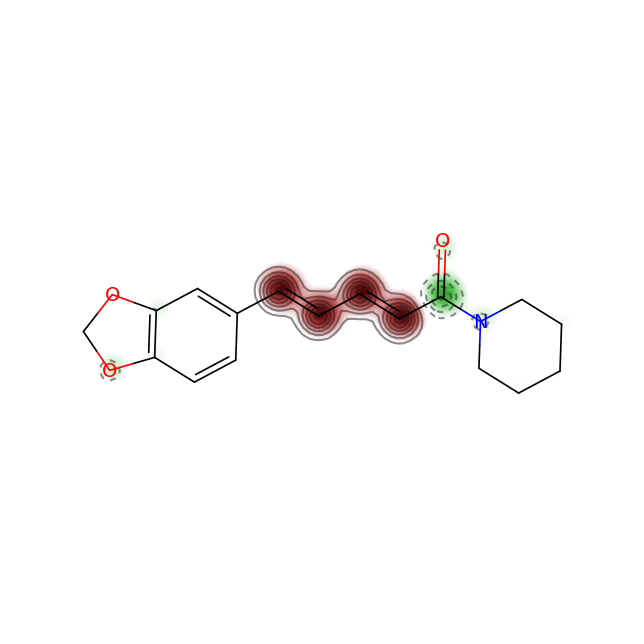 |
| 6. | Skin irritation and corrosion | Negative (-) | 70 | 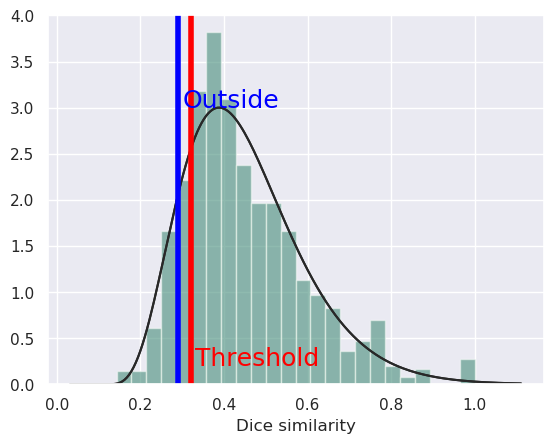 | 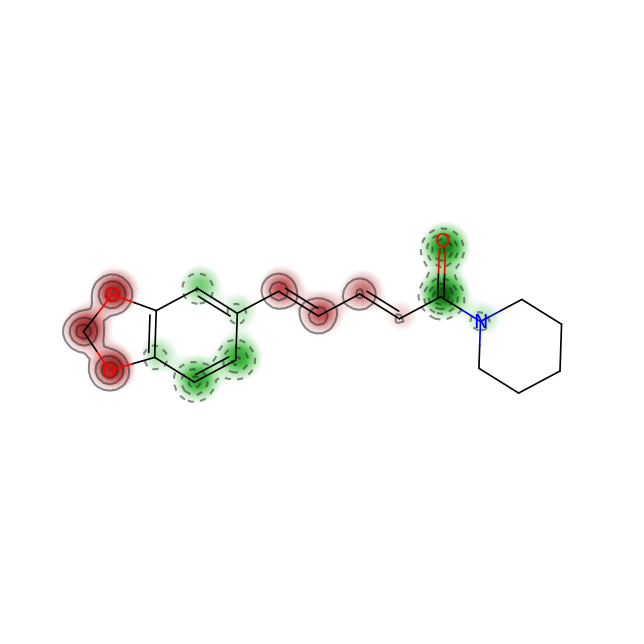 |

**Supplementary Table 4. Binding affinity values of piperine to osmoporin (OmpC) proteins of NTS**

| **Sl.No.** | **Compound** | **Binding scores** | | |
| --- | --- | --- | --- | --- |
|  |  | **Binding energy (kcal/mol)** | **Ligand efficiency** | **Inhibition constant (**µ**M)** |
| 1 | Piperine | -6.55 | -0.31 | 15.85 |

**Supplementary Table 5. MIC and MBC values of green synthesized ZnO NPs against MDR-NTS isolates**

| **ISOLATES** | | **MIC/MBC (μg/mL)** |
| --- | --- | --- |
| ***S*. Enteritidis** | **S1** | 62.5/125 |
|  | **S2** | 62.5/125 |
|  | **S3** | 62.5/125 |
| ***S*. Typhimurium** | **ST1** | 62.5/125 |
|  | **ST2** | 62.5/125 |
|  | **ST3** | 62.5/125 |

**Supplementary Table 6. *In vitro* stability of ZnO NPs at high-end temperatures (70 °C and 90 °C)**

| **ISOLATES** | **Temperatures** | | | | | |
| --- | --- | --- | --- | --- | --- | --- |
|  | **70 °C** | | | **90 °C** | | |
|  | **5 min** | **15 min** | **30 min** | **5 min** | **15 min** | **30 min** |
| **S1** | 62.5/250 | 62.5/250 | 62.5/250 | 62.5/375 | 62.5/250 | 62.5/250 |
| **S2** | 62.5/250 | 62.5/250 | 62.5/250 | 62.5/250 | 62.5/375 | 62.5/250 |
| **S3** | 62.5/375 | 62.5/250 | 62.5/375 | 62.5/375 | 62.5/375 | 62.5/375 |
| **ST1** | 62.5/250 | 62.5/250 | 62.5/250 | 62.5/250 | 62.5/250 | 62.5/375 |
| **ST2** | 125/375 | 125/375 | 125/375 | 62.5/375 | 62.5/375 | 62.5/250 |
| **ST3** | 125/375 | 125/375 | 125/375 | 62.5/250 | 62.5/375 | 62.5/375 |

**Supplementary Table 7. *In vitro* stability of ZnO NPs from piperine on exposure to trypsin, lysozyme, and proteinase- K**

| **ISOLATES** | **MIC/MBC (μg/mL)** | | | | | | | | | | | |
| --- | --- | --- | --- | --- | --- | --- | --- | --- | --- | --- | --- | --- |
|  | **PROTEINASE- K** | | | | **LYSOZYME** | | | | **TRYPSIN** | | | |
|  | **30 sec** | **5 min** | **15 min** | **30 min** | **30 sec** | **5 min** | **15 min** | **30 min** | **30 sec** | **5 min** | **15 min** | **30 min** |
| **S1** | 62.5/375 | 62.5/250 | 62.5/375 | 62.5/375 | 62.5/250 | 62.5/375 | 62.5/375 | 62.5/500 | 62.5/250 | 31.25/250 | 31.25/375 | 31.25/375 |
| **S2** | 62.5/250 | 62.5/375 | 62.5/375 | 62.5/500 | 62.5/375 | 62.5/375 | 62.5/500 | 62.5/125 | 62.5/250 | 31.25/250 | 31.25/250 | 31.25/250 |
| **S3** | 62.5/250 | 62.5/375 | 62.5/375 | 62.5/375 | 62.5/250 | 62.5/500 | 62.5/500 | 62.5/500 | 125/375 | 62.5/500 | 31.25/250 | 31.25/250 |
| **ST1** | 62.5/250 | 62.5/500 | 62.5/375 | 62.5/500 | 62.5/250 | 62.5/500 | 62.5/500 | 62.5/500 | 125/375 | 62.5/500 | 31.25/250 | 31.25/375 |
| **ST2** | 62.5/375 | 62.5/375 | 62.5/500 | 62.5/500 | 62.5/250 | 125/375 | 125/375 | 125/500 | 62.5/250 | 31.25/375 | 62.5/500 | 62.5/375 |
| **ST3** | 62.5/375 | 62.5/500 | 62.5/500 | 62.5/500 | 125/375 | 125/500 | 125/500 | 125/500 | 62.5/250 | 31.25/250 | 31.25/500 | 62.5/500 |

**Supplementary Table 8. *In vitro* stability of green synthesized ZnO NPs on exposure to the physiological concentration of cationic salts (150 m*M* NaCl and 2m*M* MgCl_2_)**

| **ISOLATES** | **MIC/MBC (μg/mL)** | |
| --- | --- | --- |
|  | **NaCl (150 m*M*)** | **MgCl_2_ (2 m*M*)** |
| **S1** | 62.5/125 | 62.5/125 |
| **S2** | 62.5/125 | 62.5/125 |
| **S3** | 62.5/125 | 62.5/125 |
| **ST1** | 62.5/125 | 62.5/125 |
| **ST2** | 62.5/125 | 62.5/125 |
| **ST3** | 62.5/125 | 62.5/125 |

**Supplementary Table 9. *In vitro* stability of green synthesized ZnO NPs on exposure to different pH (4.0, 6.0, 8.0)**

| **ISOLATES** | **MIC/MBC (μg/mL)** | | |
| --- | --- | --- | --- |
|  | **ZnO NPs- Piperine** | | |
|  | **pH : 4** | **pH : 6** | **pH : 8** |
| **S1** | 125/250 | 125/250 | 31.25/62.5 |
| **S2** | 125/250 | 125/250 | 31.25/62.5 |
| **S3** | 125/250 | 125/250 | 31.25/62.5 |
| **ST1** | 125/250 | 125/250 | 31.25/62.5 |
| **ST2** | 125/250 | 125/250 | 31.25/62.5 |
| **ST3** | 125/250 | 125/250 | 31.25/62.5 |

**Supplementary Table 10. Antibiofilm potential of ZnO NPs against MDR-NTS**

| **Bacterial strains** | **Strains** | **MIC (1X)** | |
| --- | --- | --- | --- |
|  |  | **24 h** | **48 h** |
| **MDR-*S*. Enteritidis** | **S1** | 10.563 | 40.940 |
|  | **S2** | 29.412 | 46.939 |
|  | **S3** | 18.868 | 23.034 |
| **MDR-*S*. Typhimurium** | **ST1** | 13.380 | 25.503 |
|  | **ST2** | 15.686 | 31.293 |
|  | **ST3** | 2.113 | 27.329 |


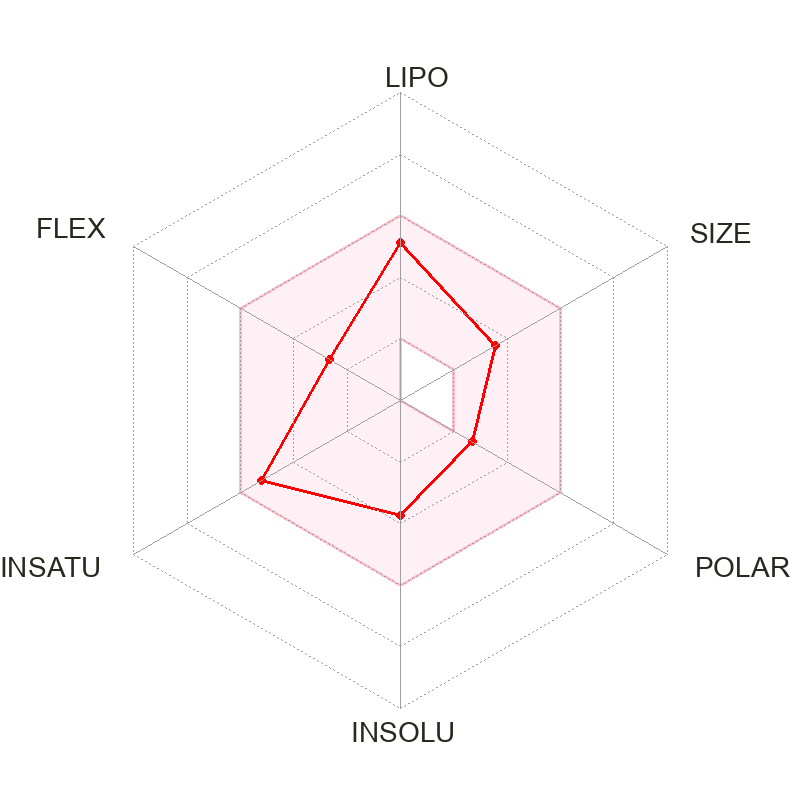


**Supplementary Fig. 1 Bioavailability radar of piperine**


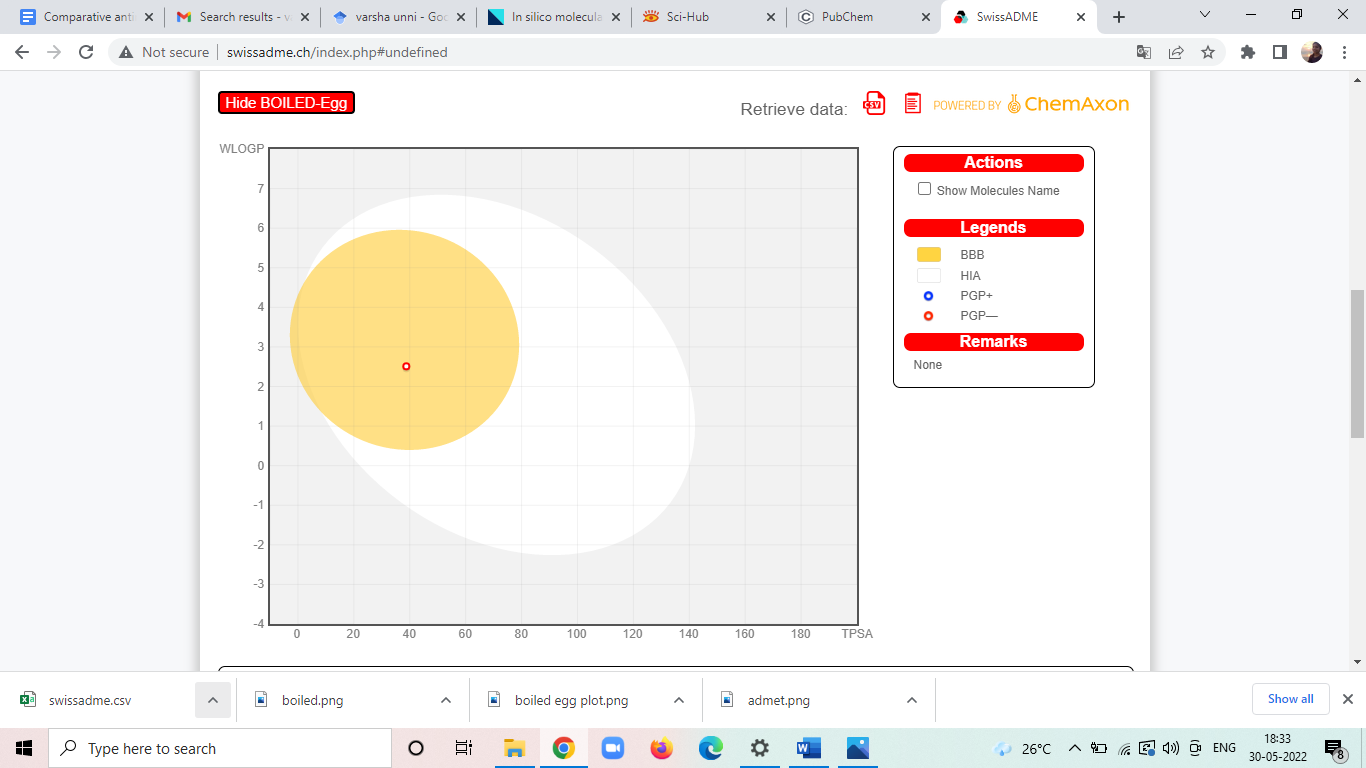


**Supplementary Fig. 2 Boiled egg graph of piperine**


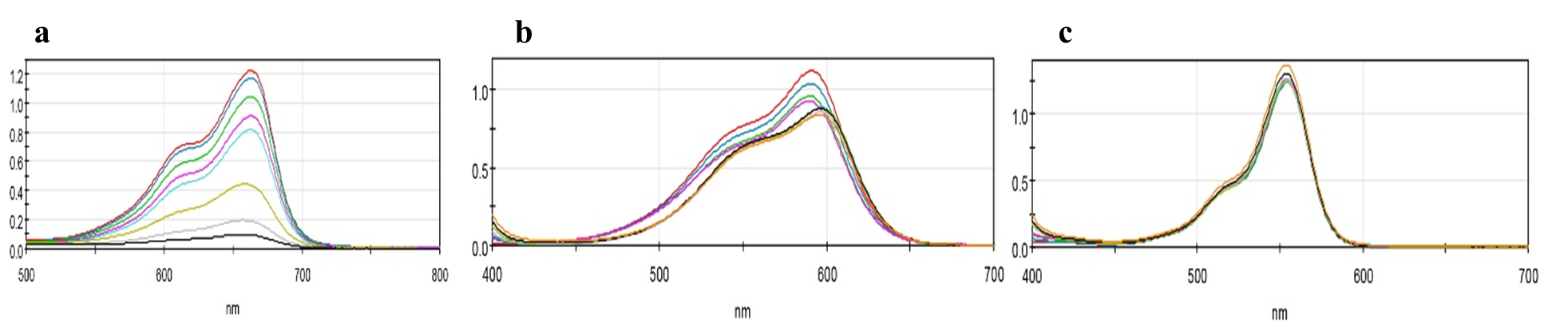


**Supplementary Fig. 3A. UV-Vis spectroscopic patterns of dye degradation of MB (a), CV (b), and RhB (c) on treatment with ZnO NPs, under sunlight**


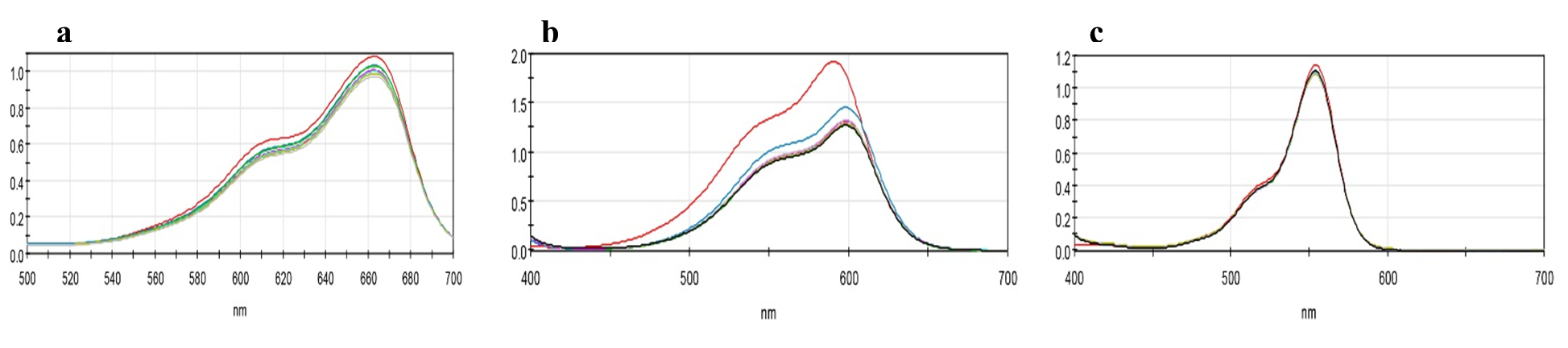


**Supplementary Fig. 3B. UV-Vis spectroscopic patterns of dye degradation of MB (a), CV (b), and RhB (c) on treatment with ZnO NPs, under LED light**


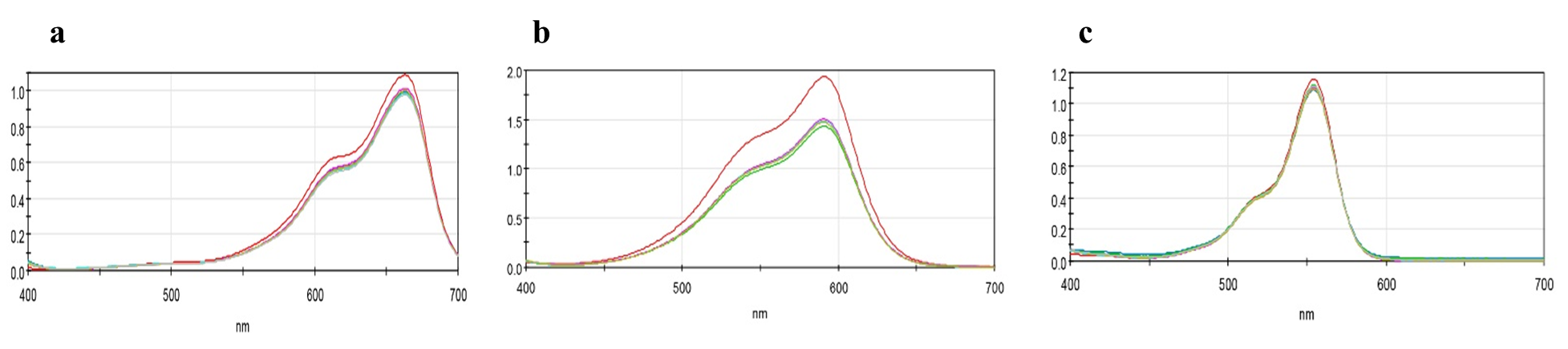


**Supplementary Fig. 3C. UV-Vis spectroscopic patterns of dye degradation of MB (a), CV (b), and RhB (c) on treatment with ZnO NPs, under UV light**
